# Unveiling mechanisms underlying kidney function changes during sex hormone therapy

**DOI:** 10.1172/JCI190850

**Published:** 2025-03-25

**Authors:** Sarah A. van Eeghen, Laura Pyle, Phoom Narongkiatikhun, Ye Ji Choi, Wassim Obeid, Chirag R. Parikh, Taryn G. Vosters, Irene G.M. van Valkengoed, Merle M. Krebber, Daan J. Touw, Martin den Heijer, Petter Bjornstad, Daniël H. van Raalte, Natalie J. Nokoff

**Affiliations:** 1 Center of Expertise on Gender Dysphoria, Department of Internal Medicine, Amsterdam UMC, Location VU University, Amsterdam, Netherlands.; 2 Amsterdam Gastroenterology Endocrinology Metabolism Research Institute, Amsterdam, Netherlands.; 3 Department of Internal Medicine, Endocrinology and Metabolism, Amsterdam UMC, Location VU University, Amsterdam, Netherlands.; 4 Department of Medicine, Division of Endocrinology, Metabolism and Nutrition, University of Washington School of Medicine, Seattle, Washington, USA.; 5 Department of Pediatrics, Section of Endocrinology, University of Colorado School of Medicine, Aurora, Colorado, USA.; 6 Division of Nephrology, Department of Internal Medicine, Faculty of Medicine, Chiang Mai University, Chiang Mai, Thailand.; 7 Division of Nephrology, Internal Medicine, Johns Hopkins School of Medicine, Baltimore, Maryland, USA.; 8 Department of Public and Occupational Health, Amsterdam University Medical Centre, Universiteit van Amsterdam, Amsterdam, Netherlands.; 9 Department of Nephrology and Hypertension, University Medical Center, Utrecht, Netherlands.; 10 Department of Clinical Pharmacy & Pharmacology, University of Groningen, University Medical Center Groningen, Groningen, Netherlands.; 11 Diabetes Center, Department of Internal Medicine, Amsterdam UMC, Location VU University, Amsterdam, Netherlands.; 12 Amsterdam Cardiovascular Sciences Research Institute, VU University, Amsterdam, Netherlands.

**Keywords:** Endocrinology, Nephrology, Chronic kidney disease, Sex hormones

## Abstract

**BACKGROUND:**

Men with chronic kidney disease (CKD) experience faster kidney function decline than women. Studies in individuals undergoing sex hormone therapy suggest a role for sex hormones, as estimated glomerular filtration rate (eGFR) increases with feminizing therapy and decreases with masculinizing therapy. However, effects on measured GFR (mGFR), glomerular and tubular function, and involved molecular mechanisms remain unexplored.

**METHODS:**

This prospective, observational study included individuals initiating feminizing (estradiol and antiandrogens; *n* = 23) or masculinizing (testosterone; *n* = 21) therapy. Baseline and 3-month assessments included mGFR (iohexol clearance), kidney perfusion (para-aminohippuric acid clearance), tubular injury biomarkers, and plasma proteomics.

**RESULTS:**

During feminizing therapy, mGFR and kidney perfusion increased (+3.6% and +9.1%, respectively; *P* < 0.05) without increased glomerular pressure. Tubular injury biomarkers, including urine neutrophil gelatinase-associated lipocalin, epidermal growth factor (EGF), monocyte chemoattractant protein-1, and chitinase 3-like protein 1 (YKL-40), decreased significantly (–53%, –42%, –45%, and –58%, respectively). During masculinizing therapy, mGFR and kidney perfusion remained unchanged, but urine YKL-40 and plasma tumor necrosis factor receptor 1 (TNFR-1) increased (+134% and +8%, respectively; *P* < 0.05). Proteomic analysis revealed differential expression of 49 proteins during feminizing and 356 proteins during masculinizing therapy. Many kidney-protective proteins were positively associated with estradiol and negatively associated with testosterone, including proteins involved in endothelial function (SFRP4, SOD3), inflammation reduction (TSG-6), and maintaining kidney tissue structure (agrin).

**CONCLUSION:**

Sex hormones influence kidney physiology, with estradiol showing protective effects on glomerular and tubular function, while testosterone predominantly exerts opposing effects. These findings emphasize the role of sex hormones in sexual dimorphism observed in kidney function and physiology and suggest new approaches for sex-specific precision medicine.

**TRIAL REGISTRATION:**

Dutch Trial Register (ID: NL9517); ClinicalTrials.gov (ID: NCT04482920).

## Abstract 2

Sex hormones seem to influence kidney function; with estradiol appearing protective, and testosterone inducing subclinical kidney stress

## Introduction

Chronic kidney disease (CKD) has become a global epidemic (1). Men generally experience faster CKD progression and higher CKD-related and cardiovascular mortality rates than women (1–7). This sexual dimorphism suggests that women may possess protective factors against CKD progression and its cardiovascular consequences. Among these, the sex hormone estradiol has been identified as a potential protective factor. Longer exposure to estradiol, resulting from earlier menarche or later menopause, has been linked to a lower risk of CKD (8, 9). Additionally, postmenopausal estradiol therapy has been shown to reduce albuminuria and lower the risk of kidney failure (10, 11). In addition to estradiol, the sex hormone testosterone also appears to play a role in kidney function; however, its effects are more complex due to its partial conversion into estradiol via aromatization, which makes it challenging to distinguish the direct effects of testosterone from converted estradiol (12). Several epidemiological studies have suggested that low testosterone concentrations are associated with impaired kidney function in men (13–15). However, this relationship does not apply to women, where lower testosterone concentrations are generally associated with higher estimated glomerular filtration rate (eGFR) (13). This sex-specific difference implies that the association between low testosterone and impaired eGFR in men may be more closely related to estradiol deficiency rather than low testosterone alone, further supporting estradiol’s potential protective role in kidney function (the terms women and men in this paragraph refer to presumed cisgender women and men).

Despite these insights, much of the current research on sex hormones and kidney function is based on epidemiological, cross-sectional, or animal studies (16). Experimental prospective data in humans remain scarce. Transgender individuals undergoing sex hormone therapy offer a unique opportunity to address this research gap, as it allows studying kidney physiology during controlled changes in sex hormone concentrations. This enables a more direct investigation of how estradiol and testosterone influence kidney function. While our previous work demonstrated that feminizing hormone therapy increases eGFR (estimated by serum creatinine and cystatin C), while masculinizing hormone therapy decreases it (17), these endogenous filtration markers are also influenced by factors unrelated to kidney function, such as body composition (18, 19). Directly measured GFR (mGFR) is therefore essential to validate these findings. Additionally, the mechanisms driving these rapid changes in kidney function observed in individuals during the first year of sex hormone therapy, particularly regarding glomerular and tubular function, and underlying molecular mechanisms, remain largely unexplored.

To address these gaps, we conducted a prospective observational study in 44 transgender individuals (23 initiating feminizing hormone therapy and 21 initiating masculinizing hormone therapy), with assessments before and 3 months after initiation of sex hormone therapy. We evaluated glomerular function by measuring GFR using iohexol clearance and assessed kidney perfusion by determining effective renal plasma flow (ERPF) via para-aminohippuric acid (PAH) clearance. Tubular function was evaluated using tubular injury biomarkers, and underlying molecular mechanisms were explored through plasma proteomics.

## Results

Baseline characteristics of the study population are shown in Table 1. Among the 23 individuals initiating feminizing hormone therapy, 16 (70%) were clinically prescribed with transdermal estradiol and 7 (30%) with oral estradiol. Sixteen individuals (70%) initiated a gonadotropin-releasing hormone (GnRH) analogue, 5 (22%) received spironolactone, 1 (4%) finasteride, and 1 (4%) did not start antiandrogen therapy. For the individuals initiating masculinizing hormone therapy, 17 (81%) were clinically prescribed with transdermal testosterone gel, and 4 individuals (19%) with testosterone injections, specifically, 1 (5%) with an intramuscular testosterone blend and 3 (14%) with subcutaneous testosterone cypionate.

### Sex hormone concentrations

**Figure 1:**
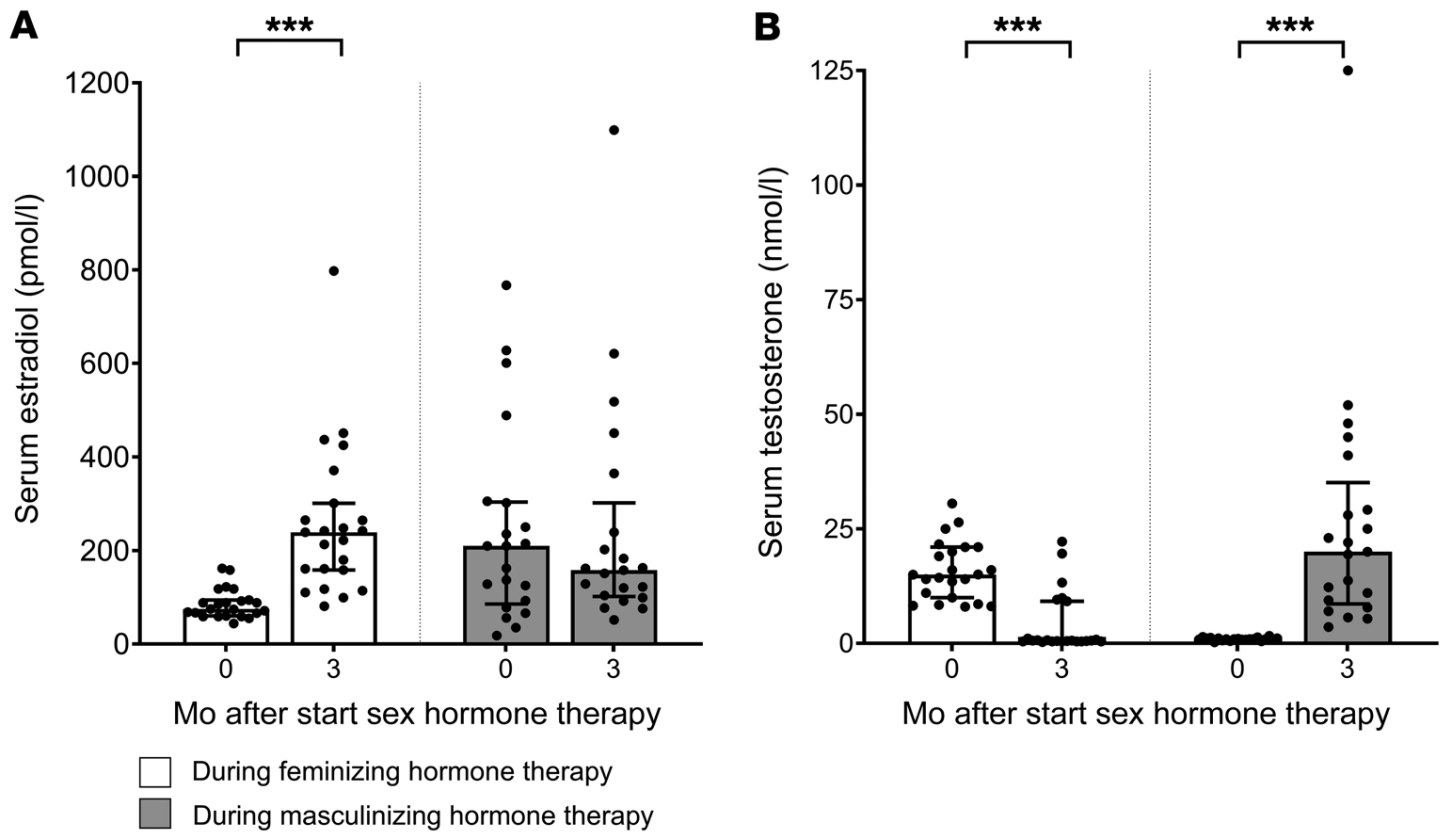
Median with IQR of serum estradiol and serum testosterone before and during 3 months of feminizing and masculinizing hormone therapy. (**A**) Total serum estradiol (pmol/L) and (**B**) total serum testosterone (nmol/L) before and during 3 months of feminizing (*n* = 23) and masculinizing (*n* = 21) hormone therapy. The differences between baseline and 3-month values were evaluated using Wilcoxon’s signed-rank test due to nonnormal distribution of the data. One participant had an unusually high serum testosterone concentration (125 nmol/L) during the 3-month study visit, despite reportedly receiving only 40.5 mg of transdermal testosterone per day, possibly due to external contamination of the gel. Consequently, this testosterone measurement was excluded from all subsequent analyses. ***P < 0.001.

In individuals undergoing feminizing hormone therapy, median estradiol concentrations increased from 75 (IQR, 60–94) to 239 pmol/L (IQR, 158–301; *P* < 0.001) after 3 months of therapy. Concurrently, testosterone concentrations decreased from 15.0 (IQR, 10.0–21.0) to 0.6 nmol/L (IQR, 0.4–9.2; *P* < 0.001; Figure 1 and Supplemental Table 1). During masculinizing hormone therapy, median estradiol concentrations remained stable (at 3 months: 158 pmol/L; IQR, 104–239; *P* = 0.54), whereas testosterone concentrations increased from 0.9 (IQR, 0.8–1.1) to 20.0 nmol/L (IQR, 9.4–29.2; *P* < 0.001; Figure 1 and Supplemental Table 1). There were no statistically significant differences in serum concentrations of estradiol by route of estradiol therapy or testosterone by route of testosterone therapy (data not shown).

One participant had an unusually high serum testosterone concentration (125 nmol/L) during the 3-month study visit despite reportedly receiving only 40.5 mg of transdermal testosterone per day (Figure 1). This is possibly due to external contamination of the gel (20). Consequently, this testosterone measurement was excluded from all subsequent analyses.

### Blood pressure, body mass index, and body composition

Feminizing hormone therapy was associated with a decrease in MAP of 3 mmHg (87 [±9] to 84 [±11] mmHg; *P* = 0.045), while MAP remained unchanged during masculinizing hormone therapy (80 [±7] to 80 [±6] mmHg, *P* = 0.60; Supplemental Table 1). BMI showed no differences during feminizing hormone therapy (25.9 [±8.5] to 26.0 [±8.3] kg/m²; *P* = 0.44), whereas BMI increased during masculinizing hormone therapy by 0.5 kg/m² (24.5 [±3.8] to 25.0 [±4.0] kg/m²; *P* = 0.005; Supplemental Table 1).

Fat-free mass remained stable during 3 months of feminizing hormone therapy (+0.4%, *P* = 0.62), but increased during masculinizing hormone therapy by 9.7% (*P* < 0.001). Similarly, fat mass showed no changes during feminizing hormone therapy (–4.6%; *P* = 0.44), while it decreased during masculinizing hormone therapy by 15.1% (*P* < 0.001; Supplemental Table 2).

### Glomerular function, kidney perfusion, and other intrakidney hemodynamic parameters

**Figure 2:**
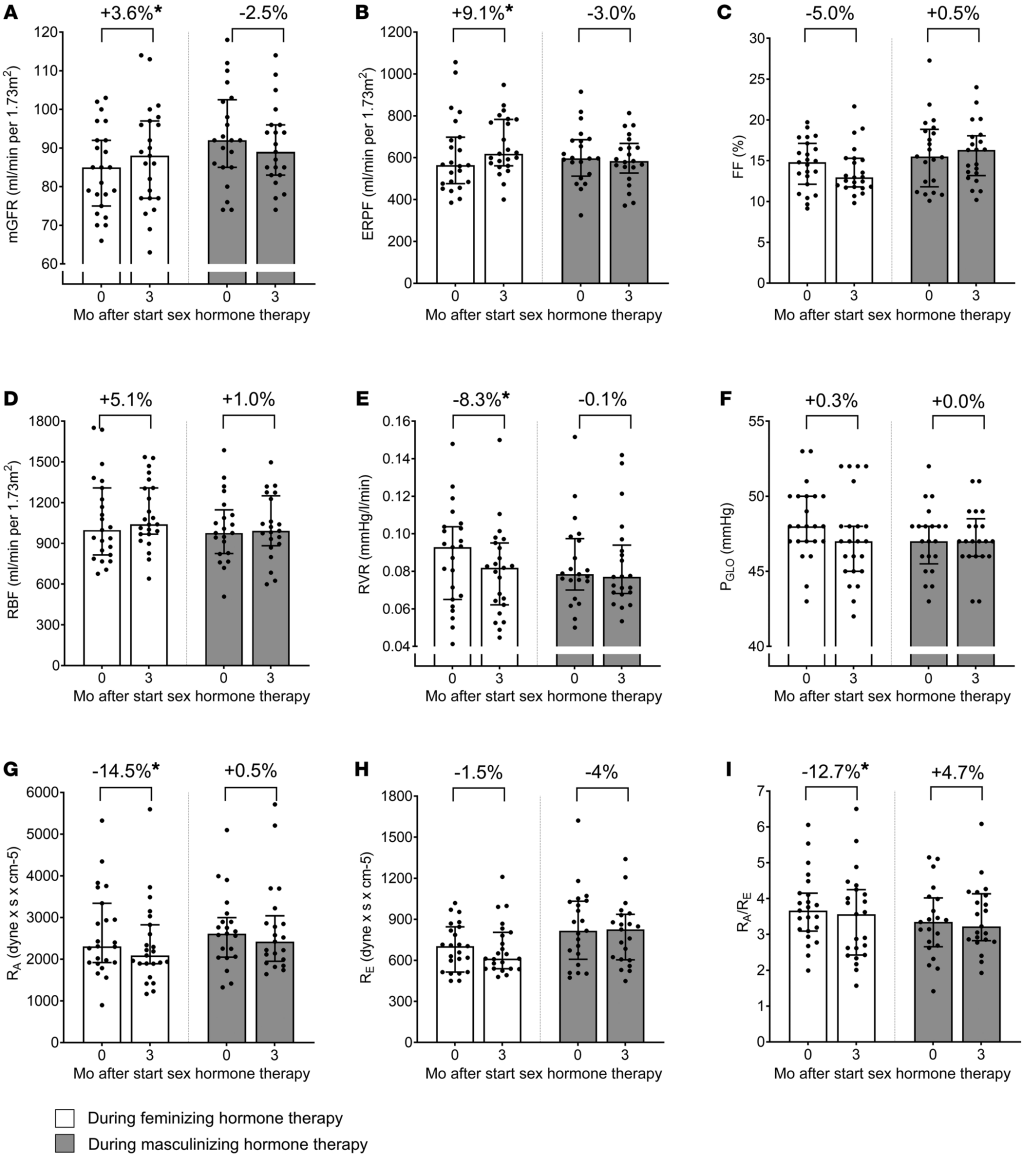
mGFR and intrakidney hemodynamic function before and during 3 months of feminizing and masculinizing therapy with percentage changes. (**A**) mGRF (ml/min per 1.72 m^2^). (**B**) ERPF (ml/min per 1.72 m^2^). (**C**) FF (%). (**D**) RBF (ml/min per 1.72 m^2^). (**E**) RVR (mmHg/L/min). (**F**) P_GLO_ (mmHg). (**G**) R_A_ (dyne x s x cm^–5^). (**H**) R_E_ (dyne x s x cm^–5^). (**I**) R_A_/R_E_. Data were collected from 23 individuals receiving feminizing hormone therapy and 21 individuals receiving masculinizing hormone therapy. Data are presented as median (IQR). *y* axis is in linear scale. *P < 0.05. Percentage changes for P_GLO_, R_A_, and R_A_/R_E_, during feminizing hormone therapy were adjusted for Δ total protein. For percentage change, variables were log transformed, and linear mixed models were applied to the log-transformed data, clustering measurements within participants. The resulting ratios, along with 95% confidence intervals, were back transformed and presented as percentage changes for comparison between baseline and 3-month measurements.

During feminizing hormone therapy, mGFR increased by 3.6% (85.0 [IQR, 75.2–92.4] to 87.9 [IQR, 77.1–96.7] mL/min per 1.73 m^2^; *P* = 0.041), and ERPF increased by 9.1% (564 [IQR, 476–698] to 619 [IQR, 561–783] mL/min per 1.73 m^2^; *P* = 0.022), while renal vascular resistance (RVR) decreased by 8.3% (*P* = 0.048; Figure 2 and Supplemental Table 3). Plasma total protein concentration decreased by 3.3% (*P* = 0.001; Supplemental Table 4) with feminizing hormone therapy, and this was adjusted for in our analysis of glomerular pressure (P_GLO_), afferent arteriole resistance (R_A_), and the afferent to efferent resistance ratio (R_A_/R_E_). R_A_ and R_A_/R_E_ decreased during feminizing hormone therapy (Figure 2; Supplemental Table 3). In contrast, masculinizing hormone therapy led to a nonsignificant decrease in mGFR (by –2.5%; 91.9 [IQR, 85.3–101.9] to 89.1 [IQR, 83.5–95.5] mL/min per 1.73 m^2^; *P* = 0.20) and ERPF (by –3.0%; 597 [IQR, 522–682] to 584 [IQR, 554–648] mL/min per 1.73 m^2^; *P* = 0.31), with other intrakidney hemodynamic parameters remaining unchanged (Figure 2; Supplemental Table 3).

Excluding participants using spironolactone and adjusting for Δ fat-free mass or Δ fat mass (where Δ represents the absolute change from baseline to 3 months) yielded similar results during feminizing hormone therapy (Supplemental Tables 5 and 6). For masculinizing hormone therapy, adjusting for Δ fat-free mass reduced the decline in mGFR and ERPF to –1.4% and –1.1%, respectively (Supplemental Table 6).

**Figure 3:**
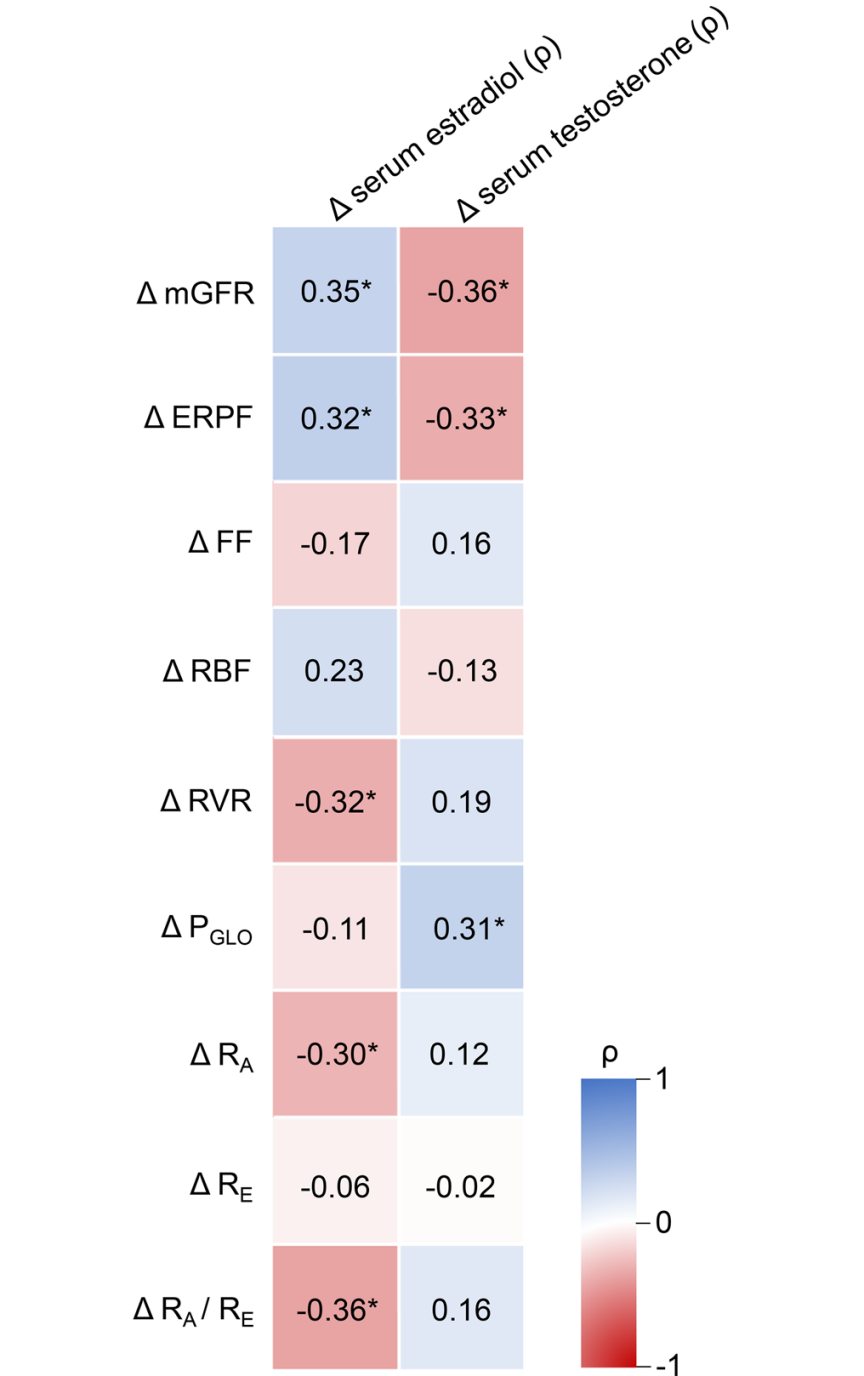
Heatmap with correlations between Δ serum estradiol and serum testosterone and Δ mGFR, ERPF and intra-kidney hemodynamic parameters. Using Spearman rank’s correlation coefficient (ρ), considering masculinizing and feminizing hormone therapy together as 1 group (*n* = 43 for correlations with serum estradiol and *n* = 42 for correlations with serum testosterone). *P < 0.05. mGFR (mL/min per 1.73 m^2^); ERPF (mL/min per 1.73 m^2^); FF (%); RBF (mL/min per 1.73 m^2^); RVR (mmHg/L/min; corrected for BSA); P_GLO_ (mmHg); R_A_ (dyne × s × cm^-5^); R_E_ (dyne × s × cm^-5^).

Considering individuals undergoing feminizing and masculinizing therapies as a single group, Δ mGFR, ERPF, RVR, R_A_, and R_A_/R_E_ ratio correlated with Δ serum estradiol (mGFR: ρ = 0.35, *P* = 0.019; ERPF: ρ = 0.32, *P* = 0.032; RVR: ρ = –0.32, *P* = 0.036; R_A_: ρ = –0.30; *P* = 0.046; R_A_/R_E_ ratio: ρ = –0.36; *P* = 0.015; Figure 3). Similarly, Δ mGFR, ERPF, and P_GLO_ correlated with Δ serum testosterone (mGFR: ρ = –0.36; *P* = 0.020; ERPF: ρ = –0.33; *P* = 0.030; P_GLO_: ρ = 0.31; *P* = 0.041; Figure 3). When considering feminizing and masculinizing hormone therapy separately, Δ mGFR was correlated with Δ estradiol during feminizing hormone therapy (ρ = 0.51, *P* = 0.014).

### Tubular function; tubular injury biomarkers

**Figure 4:**
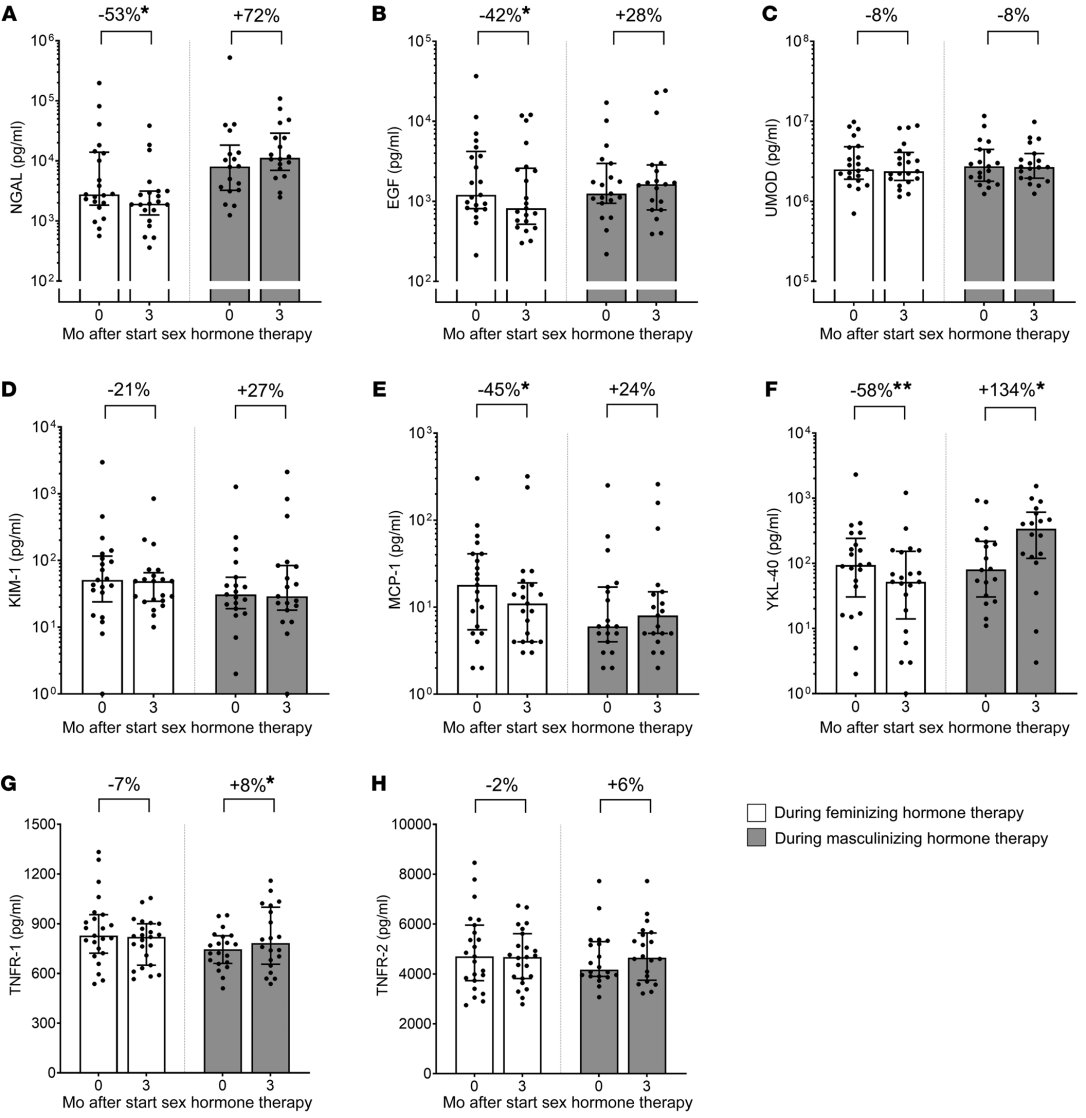
Tubular injury biomarkers before and during 3 months of feminizing and masculinizing therapy with the percentage changes. Urinary tubular injury biomarkers were collected from 21 individuals receiving feminizing hormone therapy, while plasma tubular injury biomarkers were collected from 23 individuals receiving feminizing hormone therapy. In the masculinizing hormone therapy group, urinary NGAL and YKL-40 were collected from 18 individuals, whereas other urinary tubular injury biomarkers were collected from 19 individuals. Plasma tubular injury biomarkers were obtained from 20 individuals receiving masculinizing hormone therapy. Data are presented as median (IQR). For urine tubular injury biomarkers (**A**–**F**), the *y* axis is in log scale, and for plasma tubular injury biomarkers (**G** and **H**), the *y* axis is in linear scale. Percentage changes were adjusted for Δ mGFR. For percentage change, variables were log transformed, and linear mixed models were applied to the log-transformed data, clustering measurements within participants. The resulting ratios, along with 95% confidence intervals, were back transformed and presented as percentage changes for comparison between baseline and 3-month measurements. *P < 0.05; **P < 0.01.

**Figure 5:**
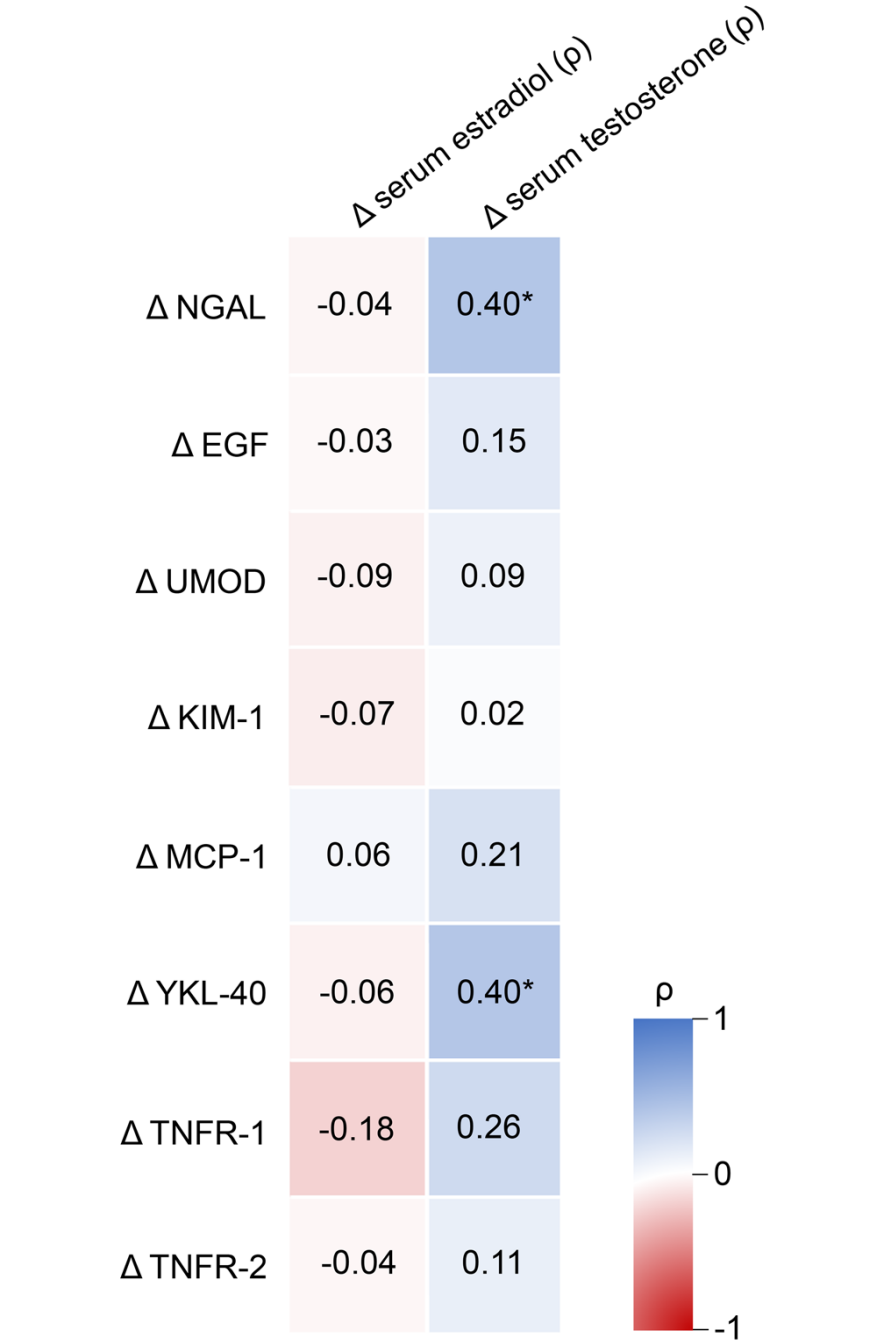
Heatmap with correlations between Δ serum estradiol and serum testosterone and Δ tubular injury biomarkers. Using Spearman’s rank correlation coefficient (ρ), considering masculinizing and feminizing hormone therapy together as 1 group (*n* = 39 for NGAL and YKL-40, *n* = 40 for EGF, UMOD, KIM-1, and MCP-1, and *n* = 43 for TNFR-1 and TNFR-2 in correlations with serum estradiol; each sample size is reduced by 1 for correlations with serum testosterone). *P < 0.05.

Figure 4 illustrates the percentage changes in known urine and plasma tubular injury biomarkers over the 3 months following sex hormone therapy initiation, adjusted for Δ mGFR. During feminizing hormone therapy, urine neutrophil gelatinase-associated lipocalin (NGAL), epidermal growth factor (EGF), monocyte chemoattractant protein-1 (MCP-1), and chitinase 3-like protein 1 (YKL-40) decreased (–53%, –42%, –45%, and –58%, respectively; *P* < 0.05). In contrast, during masculinizing therapy urine YKL-40 and plasma TNF receptor 1 (TNFR-1) increased (+134% and +8%, respectively; *P* < 0.05). Considering individuals undergoing feminizing and masculinizing therapies as a single group, Δ NGAL and YKL-40 were correlated with Δ serum testosterone (for both NGAL and YKL-40: ρ = 0.40; *P* = 0.013; Figure 5). Other biomarkers showed no significant changes or correlations (Figure 4 and Figure 5; Supplemental Table 7).

### Plasma proteomics for underlying molecular mechanisms

#### Differentially expressed proteins during feminizing hormone therapy.

**Figure 6:**
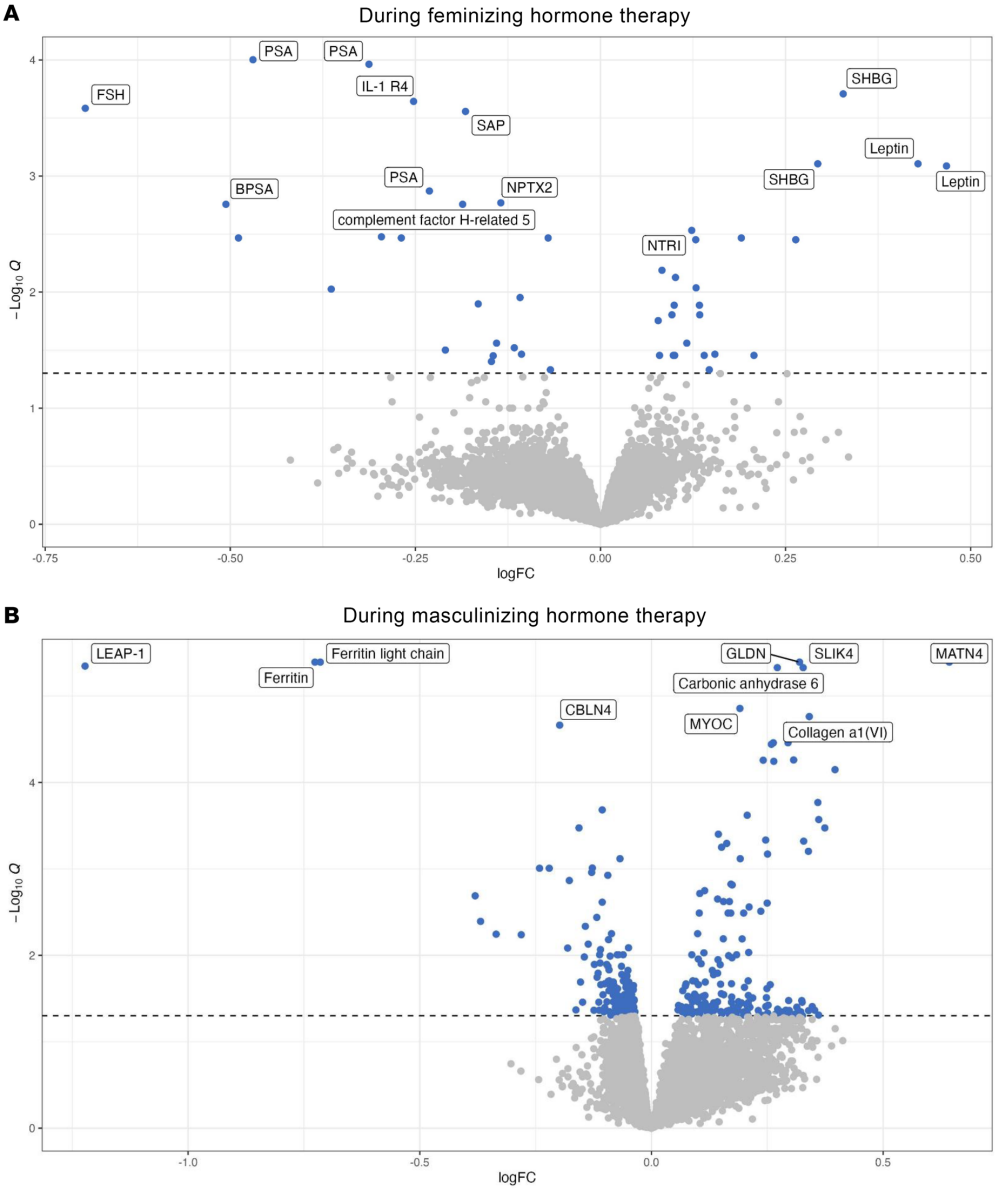
Volcano plot describing the DEPs during feminizing hormone therapy and masculinizing hormone therapy. Data were collected from 23 individuals receiving feminizing hormone therapy (**A**) and 20 individuals receiving masculinizing hormone therapy (**B**). Each dot represents an individual protein, with significantly different proteins highlighted in blue. *P* values were adjusted to maintain a false discovery rate of 5%. The top 10 proteins in each group are labeled by name. Some duplicates are present due to the use of different aptamers targeting the same or similar proteins.

Feminizing hormone therapy was associated with 49 differentially expressed proteins (DEPs). Among the top 10 DEPs, prostate-specific antigen (PSA), interleukin-1 receptor-like 1 (IL-1 R4), follicle-stimulating hormone (FSH), serum amyloid P-component (SAP), neuronal pentraxin-2 (NPTX2), benign prostate-specific antigen (BPSA), and complement factor H-related protein 5 were downregulated. Conversely, sex hormone-binding globulin (SHBG), leptin, and neurotrimin (NTRI) were upregulated (Figure 6A).

#### DEPs during masculinizing hormone therapy.

Masculinizing hormone therapy was associated with 356 DEPs. The top 10 DEPs included downregulation of ferritin light chain, ferritin, hepcidin (LEAP-1), and cerebellin-4 (CBLN4), and upregulation of matrilin-4 (MATN4), gliomedin (GLDN), SLIT and NTRK-like protein 4 (SLIK4), carbonic anhydrase 6, myocilin (MYOC), and collagen α-1(VI) chain (Figure 6B).

#### Individual proteins associated with Δ measured glomerular filtration rate.

**Figure 7:**
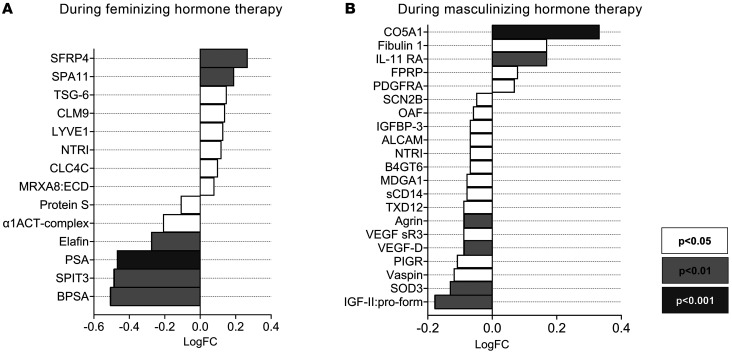
log fold changes of the identified DEPs that associated with Δ mGFR during feminizing and masculinizing hormone therapy. Data were collected from 23 individuals receiving feminizing hormone therapy (**A**) and 20 individuals receiving masculinizing hormone therapy (**B**). The listed proteins are the DEPs during feminizing or masculinizing hormone therapy, whose changes were associated with Δ mGFR. Refer to Supplemental Figure 2 for a Venn diagram summarizing the identification of these proteins. SPA11, Serpin A11; CLC4C, C-type lectin domain family 4 member C; MXRA8:ECD, matrix-remodeling-associated protein 8:extracellular domain; α1ACT-complex, α-1-antichymotrypsin complex; SPIT3, kunitz-type protease inhibitor 3; FPRP, prostaglandin F2 receptor negative regulator; SCN2B, sodium channel subunit β -2; OAF, out at first protein homolog; ALCAM, CD166 antigen; MDGA1, MAM domain-containing glycosylphosphatidylinositol anchor protein 1; sCD14, monocyte differentiation antigen CD14, soluble; TXD12, thioredoxin domain-containing protein 12; VEGF SR3, vascular endothelial growth factor receptor 3; PIGR, polymeric immunoglobulin receptor; IGF-II:Pro form, insulin-like growth factor II:Pro form.

In the full cohort, considering individuals undergoing both feminizing and masculinizing therapies as a single group, Δ mGFR correlated with changes in 385 proteins (the top 10 are shown in Supplemental Figure 1). When the analysis was narrowed to only the DEPs associated with feminizing or masculinizing hormone therapy, a total of 34 DEPs were found to correlate with Δ mGFR (Table 2 and Figure 7). Refer to Supplemental Figure 2 for a Venn diagram summarizing the identification of these proteins. Among the top 10 DEPs most significantly associated with Δ mGFR, secreted frizzled-related protein 4 (SFRP4), PDGFR α (PDGFRA), TNF-inducible gene 6 protein (TSG-6), extracellular superoxide dismutase 3 (SOD3), CMRF35-like molecule 9 (CLM9), IGF-II: Proform, interleukin 11 receptor subunit α (IL-11 RA), vascular endothelial growth factor D (VEGF-D), and IGF-binding protein 3 (IGFBP3), were positively associated with Δ mGFR, while PSA was negatively associated with Δ mGFR (*P* < 0.01).

#### Individual proteins associated with changes in ERPF.

**Figure 8:**
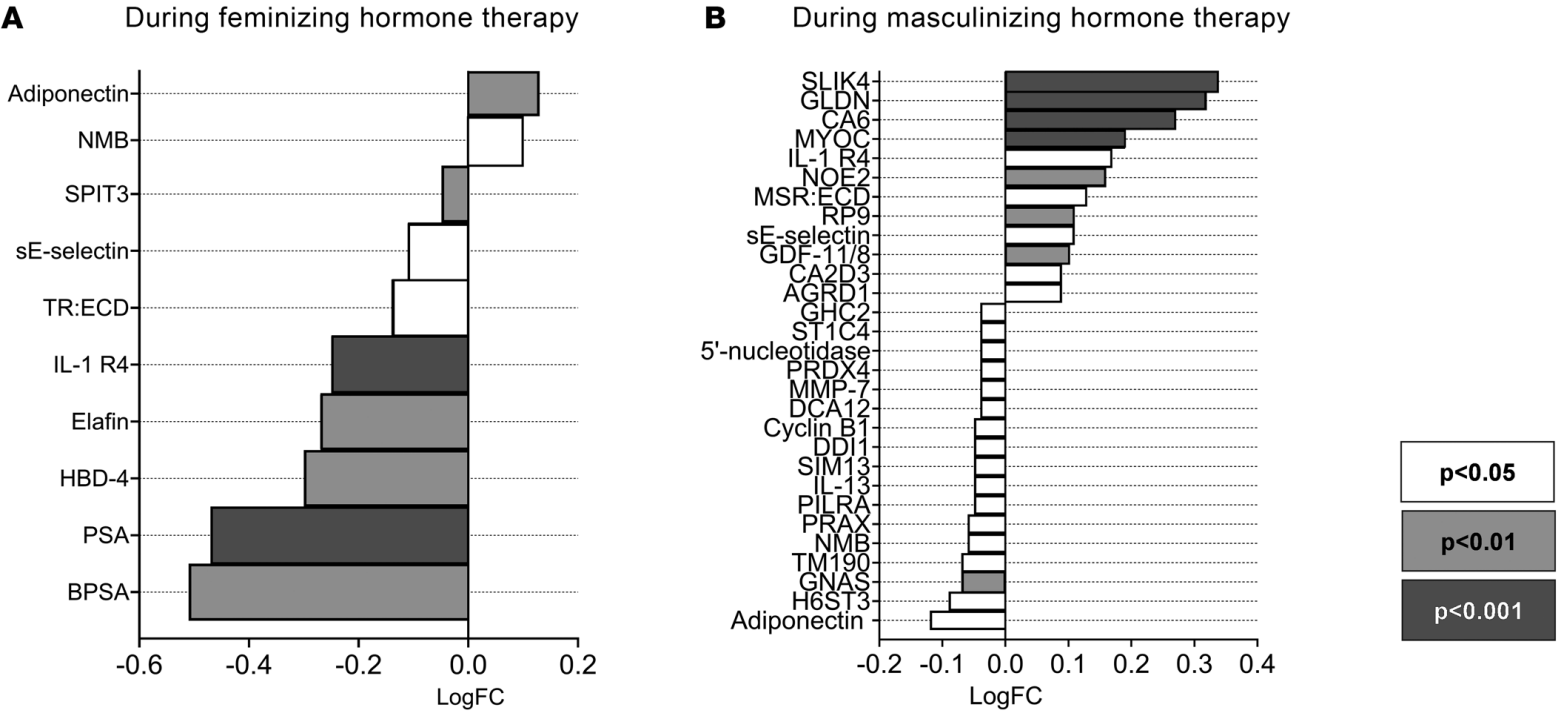
log fold changes of the identified DEPs that associated with ERPF during feminizing and masculinizing hormone therapy. Data were collected from 23 individuals receiving feminizing hormone therapy (**A**) and 20 individuals receiving masculinizing hormone therapy (**B**). The listed proteins are the DEPs during feminizing or masculinizing hormone therapy, whose changes were associated with Δ ERPF. Refer to Supplemental Figure 2 for a Venn diagram summarizing the identification of these proteins. NMB, neuromedin-B; TR:ECD, transferrin receptor protein 1:extracellular domain; HBD-4, β-defensin 104; CA6, carbonic anhydrase 6; NOE2, noelin-2; MSR:ECD, macrophage scavenger receptor: extracellular domain; RP9, retinitis pigmentosa 9 protein; CA2D3, voltage-dependent calcium channel subunit α-2/δ-3; AGRD1, adhesion G protein–coupled receptor D1; GHC2, mitochondrial glutamate carrier 2; ST1C4, sulfotransferase 1C4; SIM13, small integral membrane protein 13; PRAX, periaxin; GNAS, guanine nucleotide-binding protein G(s) subunit α isoforms; H6ST3, heparan-sulfate 6-O-sulfotransferase 3.

Δ ERPF correlated with 265 proteins, with the top 10 shown in Supplemental Figure 1. When the analysis was restricted to DEPs associated with feminizing or masculinizing hormone therapy, 35 DEPs were found to correlate with Δ ERPF (Table 3 and Figure 8). Refer to Supplemental Figure 2 for a Venn diagram summarizing the identification of these proteins. Among these, the top 10 DEPs most significantly associated with Δ ERPF were DDB1- and CUL4-associated factor 12 (DCA12), cyclin B1, transmembrane protein 190 (TM190), protein DDI1 homolog 1 (DDI1), and adiponectin, which all showed positive associations with Δ ERPF (*P* < 0.05). PSA, matrix metalloproteinase-7 (MMP-7), growth/differentiation factor 11/8 (GDF-11/8), IL-1 R4, and soluble E-selectin (sE-selectin) were negatively associated with Δ ERPF.

#### Ingenuity pathway analysis to identify pathways of interest.

Ingenuity Pathway Analysis was performed using the SOMAScan assay protein set as the reference. Feminizing hormone therapy resulted in 61 differentially expressed pathways, with most of the top 10 pathways (Supplemental Figure 3) being downregulated, particularly those involved in protein synthesis and amino acid metabolism. In contrast, masculinizing hormone therapy resulted in 117 differentially expressed pathways, with most of the top 10 pathways (Supplemental Figure 3) being upregulated, notably those related to extracellular matrix (ECM) remodeling, tissue remodeling, and immune and inflammatory responses.

In the full cohort (considering individuals undergoing both feminizing and masculinizing hormone therapy as 1 group), Δ mGFR was correlated with the up- or downregulation of 81 pathways. When the analysis was restricted to the differentially expressed pathways during feminizing or masculinizing hormone therapy, 27 pathways were found to correlate with Δ mGFR. Refer to Supplemental Figure 4 for a Venn diagram summarizing the identification of these pathways. Pathways that were positively correlated with Δ mGFR were related to ECM and structural organization (glycosaminoglycan metabolism, ECM organization, and collagen degradation); development (transcriptional regulatory networks in embryonic stem cells and the hairy/enhancer-of-split related with tyrosine, arginine, proline, tryptophan motif protein 1 [HEY1] signaling pathway); cellular signaling (role of JAK2 in hormone-like cytokine signaling); pulmonary signaling pathways (pulmonary healing signaling pathway and pulmonary fibrosis idiopathic signaling pathway); cell adhesion (focal adhesion kinase [FAK] signaling); growth factor and metabolic regulation (regulation of IGF transport and uptake by IGFBPs); immunoregulation (T helper 1 [Th1] pathway, neutrophil degranulation, and immunoregulatory interactions between a lymphoid and a nonlymphoid cell); and posttranslational modifications (posttranslational protein phosphorylation; Supplemental Table 8). Pathways that were negatively correlated with Δ mGFR were related to regulation of lipid metabolism (liver X receptor and retinoid X receptor [LXR/RXR] activation); translation and protein targeting (signal recognition particle–dependent [SRP-dependent] cotranslational protein targeting to membrane, eukaryotic translation initiation, and eukaryotic initiation factor 2 [EIF2] signaling); inflammation (acute phase response signaling and role of osteoclasts in rheumatoid arthritis signaling pathway); cancer signaling (colorectal cancer metastasis signaling); angiogenesis (purinergic receptor Y [P2Y] signaling pathway); and JAK/STAT signaling (Supplemental Table 8).

In the full cohort (considering individuals undergoing both feminizing and masculinizing hormone therapy as 1 group), Δ ERPF was correlated with the up- or downregulation of 74 pathways. When the analysis was restricted to the differentially expressed pathways during feminizing or masculinizing hormone therapy, 15 pathways were found to correlate with Δ ERPF. Refer to Supplemental Figure 4 for a Venn diagram summarizing the identification of these pathways. Pathways that were positively correlated with Δ ERPF included those related to growth factor and metabolic regulation (regulation of IGF transport and uptake by IGFBPs), cell response to hypoxia (HIF1α signaling), immunity (immunoregulatory interactions between a lymphoid and a nonlymphoid cell), development (role of JAK2 in hormone-like cytokine signaling), posttranslational modifications (posttranslational protein phosphorylation), regulation of epithelial-mesenchymal transition (regulation of the epithelial-mesenchymal transition by growth factors pathway), and cardiac hypertrophy signaling (Supplemental Table 9). Pathways negatively correlated with Δ ERPF were related to cell-cycle regulation (mitotic roles of polo-like kinase; Supplemental Table 9).

For detailed proteomic data, refer to Supplemental Data Files 1–7.

## Discussion

This prospective, observational study examined changes in kidney function during sex hormone therapy in transgender individuals, focusing on glomerular function (mGFR), kidney perfusion (ERPF), tubular function (kidney injury biomarkers), and underlying molecular mechanisms (plasma proteomics). Our findings reveal that feminizing hormone therapy is associated with increased mGFR and ERPF, with no increase in P_GLO_ and decreased tubular injury biomarkers, such as urine NGAL, MCP-1, and YKL-40. Conversely, masculinizing hormone therapy appears to induce subclinical kidney stress, as evidenced by elevated tubular injury biomarkers such as urine YKL-40 and plasma TNFR-1, without significant changes in mGFR or ERPF.

Sex differences in CKD progression are well established, with cisgender women generally experiencing slower progression compared with cisgender men. This is often attributed to the effects of sex hormones (3). Two intrakidney hemodynamic markers associated with CKD progression are an increased R_A_/R_E_ ratio and elevated P_GLO_ (21). Our study suggests that during feminizing hormone therapy, increased mGFR and ERPF, combined with a decreased R_A_/R_E_ ratio and unchanged P_GLO_, reflect enhanced kidney function without inducing glomerular hyperfiltration. These kidney hemodynamic changes suggest a vasodilatory state, likely driven by elevated estradiol and reduced testosterone. Estradiol has been shown to mediate vasodilation through both direct and endothelium-dependent mechanisms, including increased NO production and suppressed ET-1–induced vasoconstriction (22–31). In contrast, testosterone exerts more complex effects, demonstrating both vasodilatory and vasoconstrictive properties, with vasoconstriction largely mediated via ET-1–induced mechanisms and renin-angiotensin-aldosterone system (RAAS) activation (28–32).

The proteomic analysis in this study further emphasizes the role of endothelium-dependent vasodilation in sex hormone–associated changes in kidney function. We identified several kidney-protective proteins involved in endothelium-dependent vasodilation, which positively correlated with Δ mGFR. Among these, SFRP4 increased during feminizing hormone therapy, whereas SOD3, VEGF-D, agrin, IGF-II: Proform, IGFBP3, and vaspin decreased during masculinizing hormone therapy. The changes in these proteins seem to be largely driven by sex hormones, as all showed negative correlations with Δ testosterone, and, except for vaspin, positive correlations with Δ estradiol. SFRP4, an inhibitor of the Wnt/β-catenin signaling pathway, has been associated with reduced diabetic nephropathy in type 2 diabetes (33). This protective effect may stem from its ability to suppress kidney fibrosis (34) and its possible potential to decrease afferent arteriole sensitivity to ET-1 through Wnt inhibition, thereby promoting vasodilation (35). SOD3, an antioxidant enzyme, reduces extracellular oxidative stress, thereby mitigating kidney fibrosis and slowing CKD progression (36, 37). In rodent studies, it has also been shown to enhance renal blood flow (RBF) after ischemic injury, potentially by increasing NO bioavailability (37–40). Furthermore, VEGF-D, primarily involved in angiogenesis and lymphangiogenesis, also induces vasodilation by activating VEGF receptor 2, which stimulates NO production via eNOS signaling (41, 42). Agrin, a proteoglycan in the glomerular basement membrane, enhances VEGF receptor 2 function, further promoting vasodilation through eNOS signaling (43). Similarly, IGF-II: Proform, and insulin-like growth factor-binding protein 3 (IGFBP-3) contribute to endothelium-dependent vasodilation. IGFs, like VEGFs, stimulate NO production, while IGFBP-3 modulates IGF-II bioavailability, likely influencing mGFR indirectly (44–46). Vaspin, an adipokine primarily secreted by visceral adipose tissue with higher concentrations in women, also enhances NO bioavailability by stimulating NO synthase activity (47, 48), which could contribute to its protective role in kidney function, particularly in preventing nephropathy in type 2 diabetes (49).

Additionally, adiponectin, which increased during feminizing hormone therapy and decreased during masculinizing hormone therapy, demonstrated a positive correlation with Δ ERPF. The change in adiponectin seems primarily driven by sex hormones, with adiponectin showing positive correlations with Δ estradiol and negative correlations with Δ testosterone. Adiponectin is an adipokine that is negatively correlated with obesity and has, like vaspin, higher concentrations in women, partly due to a lower proportion of visceral fat and a higher proportion of subcutaneous fat (50–52). Previous studies in transgender individuals have similarly highlighted adiponectin’s regulation by sex hormones (53–55). Adiponectin plays an important role in vascular and kidney health by enhancing eNOS, attenuating the RAAS (56–59), and exhibiting kidney-specific antiinflammatory and antifibrotic properties (60). In diabetic rats with adiponectin overexpression, reduced ET-1 expression was observed, which may contribute to its glomerular protective effects, as well as provide tubular protection by alleviating endoplasmic reticulum stress and apoptosis through ET-1 suppression (28, 58). Elevated adiponectin concentrations also correlate with improved endothelial function in individuals with CKD (61, 62). In contrast to adiponectin, IL-1 R4 and sE-selectin, both proteins involved in endothelial dysfunction, decreased with feminizing hormone therapy and increased with masculinizing hormone therapy. Both IL-1 R4 and sE-selectin were negatively correlated with Δ ERPF and inversely correlated with Δ estradiol, while demonstrating positive associations with Δ testosterone. Soluble IL-1 R4 (or sST2) can prevent IL-33 from binding to membrane-bound IL-1 R4, neutralizing its beneficial antifibrotic and antiinflammatory effects and attenuating endothelial NO production (63–65). Elevated soluble IL-1 R4 is associated with increased CKD risk and impaired endothelial function, assessed with flow-mediated dilation (FMD) (66–69). Similarly, sE-selectin, a cell-adhesion molecule involved in inflammation and endothelial dysfunction, also correlated negatively with FMD (70). Furthermore, elevated sE-selectin concentrations are associated with CKD (70, 71).

In summary, the kidney-protective effects of proteins such as SFRP4, SOD3, VEGF-D, agrin, IGF-II: Proform, IGFBP3, vaspin, and adiponectin are primarily mediated through endothelium-dependent vasodilation. These proteins increased during feminizing hormone therapy and/or decreased during masculinizing hormone therapy. Conversely, proteins such as IL-1 R4 and sE-selectin, both markers of endothelial dysfunction and inflammation, showed reductions with feminizing hormone therapy and elevations with masculinizing hormone therapy. These findings offer valuable insights into the molecular mechanisms underlying the effects of elevated estradiol and/or reduced testosterone on glomerular function and kidney perfusion, emphasizing the potential role of endothelium-dependent vasodilation.

Beyond proteins involved in vasodilation, additional proteins and pathways involved in kidney inflammation and fibrosis were associated with changes in kidney function during sex hormone therapy. TSG-6, a protein with antiinflammatory and antifibrotic properties, increased during feminizing hormone therapy and was positively correlated with Δ mGFR (72–74). Moreover, IL-13, an anti-inflammatory cytokine (75), peroxiredoxin-4 (PRDX4), an antioxidant (76), paired immunoglobulin-like type 2 receptor α (PILRA), an inhibitory receptor that negatively regulates neutrophil infiltration during inflammation (77), and cyclin B1, a cell-cycle progression promoter important for preventing fibrosis and promoting tissue repair in the kidneys (78) all decreased during masculinizing hormone therapy and were positively associated with Δ ERPF. Additionally, the JAK/STAT signaling pathway, known for its role in kidney inflammation and fibrosis (79), was downregulated during feminizing hormone therapy and negatively associated with Δ mGFR. Furthermore, proteins and pathways related to the ECM and structural integrity, such as the proteins collagen type V α 1 chain (CO5A1), β-1,4-galactosyltransferase 6 (B4GT6), and lymphatic vessel endothelial hyaluronic acid receptor 1 (LYVE1), and the pathways glycosaminoglycan metabolism, ECM organization, and collagen degradation were positively associated with Δ mGFR. Additionally, MMP-7, an endopeptidase that degrades a broad range of ECM substrates and serves as a key regulator of kidney fibrosis, was negatively correlated with Δ ERPF (80). These findings collectively suggest that changes in kidney function during sex hormone therapy, in addition to endothelium-dependent vasodilation, may also be influenced by the structural organization of the ECM as well as by inflammatory and fibrotic factors, as summarized in Table 4.

In addition to glomerular and proteomic changes, we also observed substantial changes in several tubular injury biomarkers during both feminizing and masculinizing hormone therapy. These changes support the idea that sex hormones affect kidney function through both glomerular and tubular mechanisms. Markers of tubulointerstitial inflammation, such as urinary MCP-1 and YKL-40, decreased during feminizing hormone therapy, indicating a reduction in kidney inflammation (81, 82). Urinary NGAL, a marker of distal tubular damage, decreased with feminizing hormone therapy (83). However, urinary EGF, a marker of epithelial integrity and repair that serves as a protective biomarker for tubular injury, also decreased (84). During masculinizing hormone therapy, urinary YKL-40 and plasma TNFR-1, both inflammatory biomarkers, increased (85). Interestingly, changes in YKL-40, as well as in NGAL, were correlated with changes in serum testosterone concentrations, suggesting a potential role for testosterone in modulating tubular stress.

This study has several strengths, including the use of gold-standard methods to assess mGFR and ERPF and the integration of proteomics analysis, which not only strengthened the rigor of our findings but also provided valuable mechanistic insights for future research. However, several limitations must also be considered. First, none of the participants had CKD, which limits our insights into the effects of sex hormone therapy in this population. Second, the 3-month follow-up restricts our ability to assess long-term impacts. Third, participants were on varying hormone regimens, as these were clinically prescribed based on availability in each country and local practices. These varied sex hormone therapy regimens among participants were not analyzed separately due to insufficient power. Fourth, we did not measure urinary albumin-to-creatinine ratio (uACR), as all participants at baseline had negative results for albuminuria on dipstick screening at baseline. We could therefore not determine the potential effects of sex hormones on uACR. Fifth, the median estradiol concentration in our study during feminizing hormone therapy was 239 pmol/L, which is below the Endocrine Society’s recommended target range of 367–734 pmol/L (100–200 pg/mL). This difference stems from the lower target range used in local protocols, which is based on maintaining a minimum estradiol concentration above 182 pmol/L to prevent bone mineral density loss during feminizing hormone therapy and on the lack of evidence that higher estradiol concentrations enhance breast development or result in more pronounced feminine changes in body composition among transgender adults (86, 87). Notably, the observed effects on kidney function may be even more pronounced at higher serum estradiol concentrations. Furthermore, the timing within the menstrual cycle can influence serum estradiol concentrations. However, we were unable to schedule the baseline visit at the same point in the cycle for each participant starting masculinizing hormone therapy, as the baseline assessment coincided with routine care appointments. Additionally, while it was also a strength, conducting the study across 2 centers could introduce potential variability in kidney testing and laboratory procedures, although all protocols were intentionally harmonized. Lastly, while we advised participants to adhere to a specific diet to minimize variability, adherence was not monitored, potentially affecting the results. Despite these limitations, the use of participants as their own controls helped mitigate some of the variability. Future research should focus on validating these findings in larger cohorts, including individuals with CKD, to better understand the long-term effects of sex hormone therapy on kidney function.

In conclusion, this study of 44 transgender adults revealed that feminizing hormone therapy is associated with increased mGFR and ERPF, without increased P_GLO_, likely due to enhanced afferent vasodilation. In contrast, masculinizing hormone therapy appeared to induce subclinical kidney stress, as evidenced by elevated kidney injury biomarkers despite no significant changes in mGFR or ERPF within the 3-month time frame. Proteomic data highlight the involvement of endothelium-dependent vasodilation, inflammation, fibrosis, and ECM organization in these sex hormone–associated changes in kidney function. These findings underscore the need for further research into the impact of sex hormones on kidney function, including studies that incorporate experimental models to confirm the suggested mechanistic pathways, which could lead to the development of sex-specific precision medicine strategies.

## Methods

### Sex as a biological variable

This study included 23 individuals assigned male at birth and 21 assigned female at birth, assessed before and 3 months after initiating sex hormone therapy. Changes in outcomes during sex hormone therapy were analyzed separately for each group. Additionally, we examined correlations between outcomes and sex hormone concentrations across all participants combined.

### Study design

The Kidney Function in People Receiving Gender Affirming Hormone Therapy (KNIGHT) study is a prospective, observational cohort study which was conducted from April of 2021 to June of 2023 at 2 sites: Amsterdam University Medical Center (Amsterdam UMC; the Netherlands) and the University of Colorado Anschutz Medical Campus (CU-AMC, United States). The study was registered at the Dutch Trial Register (ID: NL9517) and ClinicalTrials.gov (ID: NCT04482920).

### Sample size calculation, participant recruitment, and eligibility

Sample size determination was based on the primary objective of the KNIGHT study: changes in mGFR. Considering a mean difference of 10 mL/min per 1.73m² with a standard deviation of 15 mL/min per 1.73m², 80% statistical power, and a 5% significance level, the study aimed to include 20 individuals undergoing feminizing hormone therapy and 20 undergoing masculinizing hormone therapy. To account for potential dropouts and to ensure the desired sample size, additional participants were enrolled.

Recruitment was conducted through Amsterdam UMC’s center of expertise on gender dysphoria and CU-AMC’s clinical programs serving transgender individuals. Eligible participants were aged 17 to 40 or fewer years with confirmed diagnosis of gender dysphoria, who were scheduled to initiate sex hormone therapy within 1 month of enrollment. Exclusion criteria included cognitive, psychiatric, or physical impairments interfering with study procedures, prior sex hormone use, antiandrogen use or gonadectomy, pregnancy, concurrent treatment study involvement, antihypertensive medication use, existing kidney disease or diabetes, uncontrolled hypertension, cardiovascular disease history, or iodine-related allergies. A total of 44 participants completed both study visits and kidney assessments (Supplemental Figure 5): 23 underwent feminizing hormone therapy (16 from Amsterdam UMC and 7 from CU-AMC) and 21 underwent masculinizing hormone therapy (14 from Amsterdam UMC and 7 from CU-AMC).

### Treatment protocols

Sex hormone therapy was clinically prescribed and administered according to standard local protocols. At Amsterdam UMC, feminizing hormone therapy included 4 mg daily oral estradiol or 100 mcg per 24 hour transdermal patch estradiol, and a GnRH analogue, triptorelin intramuscular injection at 3.75 mg once every 4 weeks, as an antiandrogen. Masculinizing hormone therapy involved 40.5 mg transdermal testosterone gel once daily or intramuscular testosterone with a blend of 30 mg of testosterone propionate, 60 mg of phenylpropionate, 60 mg of isocaproate, and 100 mg of decanoate once every 3 weeks. At CU-AMC, feminizing hormone therapy included 0.5–1 mg daily oral estradiol at initiation or 50 mcg per 24 hours transdermal estradiol patch at initiation, and antiandrogen therapy with either 100–200 mg daily spironolactone or 1.25 mg once daily finasteride. Masculinizing hormone therapy included 20.25–40.5 mg once daily transdermal testosterone gel or 20–30 mg once weekly subcutaneous testosterone cypionate. Treatment adherence was monitored during routine care visits.

### Study procedures

Participants attended 2 visits: one before and one 3 months after initiating sex hormone therapy. Three days prior to each study visit, participants followed specific dietary guidelines and avoided strenuous activity, alcohol, and caffeine (see Supplemental Figure 6 for details). During the study visits, intravenous catheters were placed for infusions and blood sampling. Kidney function and hemodynamics were assessed using iohexol and PAH infusions, with blood and urine samples collected at specific intervals. Blood pressure and heart rate were measured, and bioimpedance analysis was conducted to assess body composition (fat mass and fat-free mass). Study procedures are described in more detail in Supplemental Figure 6.

### Outcome measures

The primary endpoints included changes in mGFR and ERPF, determined using gold-standard methods: plasma iohexol clearance for mGFR and plasma PAH clearance for ERPF. mGFR and ERPF were calculated by dividing the infusion rate by the steady-state plasma concentrations of iohexol and PAH, respectively. The steady-state plasma concentration was obtained by averaging the 3 measured plasma concentrations during kidney testing. mGFR and ERPF were adjusted for body surface area (BSA), with BSA calculated as 0.024265 × height^0.3964^ × weight^0.5378^.

The secondary endpoints included changes in intrakidney hemodynamic parameters, tubular injury biomarkers, plasma proteomics, body composition measurements, and blood pressure. Intrakidney hemodynamic parameters included RBF, calculated by dividing ERPF by (1 – hematocrit); filtration fraction (FF), calculated as mGFR divided by ERPF; and RVR, calculated as mean arterial pressure (MAP) divided by RBF. Additional intrakidney hemodynamic measures including P_GLO_ and R_A_ and R_E_ were estimated using the Gomez equations (see Supplemental Methods for details) (88).

### Laboratory measurements

#### Urine and plasma kidney biomarkers.

Urine and plasma biomarkers were measured on the Meso Scale Discovery (MSD) QuickPlex SQ120 platform (Meso Scale Diagnostics). MSD utilizes electrochemiluminescence detection and is a sandwich immunoassay organized in a patterned array format allowing for assay multiplexing. Plasma TNFR-1 and -2 were measured in samples collected at baseline. Urinary biomarkers of tubular injury (NGAL, EGF, uromodulin [UMOD], kidney injury molecule-1 [KIM-1], MCP-1, and YKL-40) were measured in fasting urine samples for the Colorado site and in 120-minute urine samples (120 minutes after the start of iohexol and PAH infusion) for the Amsterdam site, at both study visits.

#### Proteomics.

Plasma protein concentrations were measured in samples collected at baseline and at 3-month follow up using the SOMAscan 7K Proteomic platform (SomaLogic Inc.) at Washington University, St. Louis, Missouri, USA. Internal controls were run with each sample and normalized for intra- and interplate variation. The SOMAScan 7K platform comprises 7,604 aptamers corresponding to 6,596 human proteins (89).

#### Sex hormones.

At Amsterdam UMC, total estradiol concentrations were determined using liquid chromatography–tandem mass spectrometry (LC-MS/MS), with an interassay coefficient of variation (CV) of 7% and a limit of quantification (LOQ) of 5.45 pg/ml. Total testosterone concentrations were measured using LC-MS/MS, with an interassay CV ranging from 4% to 9% and an LOQ of 0.1 nmol/L. At CU-AMC, estradiol and testosterone concentrations were also measured using LC-MS/MS at Esoterix LabCorps.

#### Kidney function.

The assays creatinine, cystatin C, iohexol, and PAH are presented in Supplemental Methods.

### Statistics

#### Baseline characteristics and clinical outcomes.

Statistical analyses were performed using STATA (version 17.0). Data distribution was assessed for normality by examining histograms and comparing the mean (±SD) with the median (IQR). Baseline and 3-month measurements were summarized as absolute numbers (*n*), percentages (%), medians with IQR or means ± SD. To compare outcomes between baseline and 3 months, individuals starting masculinizing and feminizing hormone therapy were analyzed separately. Serum estradiol and testosterone concentrations were compared using Wilcoxon’s signed-rank test due to the nonnormal distribution of the data. For BMI and blood pressure, linear mixed models were employed, assuming a normal distribution, with measurements clustered within participants.

For total protein, fat mass, fat-free mass, mGFR, ERPF, intrakidney hemodynamic parameters, and tubular injury biomarkers, which were not normally distributed, data were log transformed. Linear mixed models were applied to the log-transformed data, clustering measurements within participants. Percentage changes between baseline and 3 months were derived by back-transforming the ratios and presenting them with 95% confidence intervals. Adjustments were made for Δ plasma total protein concentration for P_GLO_, R_A_, and R_A_/R_E_ percentage changes, Δ fat-free mass and fat mass for mGFR and ERPF percentage changes, and Δ mGFR for tubular injury biomarker percentage changes, where Δ represents the absolute change from baseline to 3 months. Both adjusted and unadjusted results were reported. A sensitivity analysis for the primary outcome (mGFR and ERPF) was performed by excluding participants using spironolactone.

To assess the relationship between changes in sex hormone concentrations and clinical outcomes, Spearman’s rank correlations were used due to the nonnormal distribution. Correlations were examined between Δ serum testosterone and estradiol concentrations and Δ mGFR, ERPF, intrakidney hemodynamic parameters, and tubular injury biomarkers.

#### Plasma proteomics.

Proteomic data were analyzed using R (version 4.4.0, R Core Team, Vienna). For proteomics data, proteins were log transformed and scaled by SD (i.e., each protein measurement was divided by the SD for that protein in the sample) prior to analysis. Changes in proteins between baseline and 3 months were evaluated using linear models with moderated *t* statistics (90), separately for masculinizing and feminizing hormone therapy. *P* values were adjusted to maintain a false discovery rate of 5%.

To focus on proteins more specific to kidney function, exploratory analyses were conducted to correlate protein changes with Δ mGFR and ERPF, using Spearman’s rank correlation. Additionally, Spearman’s rank correlation was used to assess relationships between protein changes and Δ serum testosterone and estradiol concentrations. These correlations were exploratory, and adjustments for multiple testing were therefore not applied. Pathway analyses were performed using Ingenuity Pathway Analysis (QIAGEN).

#### Missing data.

One participant that underwent masculinizing hormone therapy was excluded from plasma proteomics analyses and plasma tubular injury marker analyses due to insufficient plasma collection at the 3-month time point. For urine biomarker analyses, 2 participants that underwent feminizing hormone therapy and 1 that underwent masculinizing hormone therapy were excluded due to insufficient urine collection at 3 months. Additionally, 1 participant that underwent masculinizing hormone therapy was excluded due to missing baseline urine data.

Statistical significance was defined as *P* < 0.05.

### Study approval

The study was approved by the ethics review boards of both institutions and conducted in accordance with the Declaration of Helsinki and Good Clinical Practice guidelines. All participants provided written, informed consent prior to participation.

#### Data availability.

Values for all data points in graphs are reported in the Supporting Data Values file. Data can be made available upon reasonable request.

## Author contributions

SAVE participated in methodology, project administration, conducting study visits (investigation), acquiring data, data curation, analyzing data, data visualization, and writing the original draft. LP participated in plasma proteomics, analyzing data, data visualization, and reviewing and editing the manuscript. PN participated in reviewing and editing the manuscript. YJC participated in reviewing and editing the manuscript. WO participated in biomarker selection, measurement, and interpretation, and reviewing and editing the manuscript. CRP participated in biomarker selection, measurement, and interpretation, and reviewing and editing the manuscript. TGV participated in reviewing and editing the manuscript. IGMVV participated in reviewing and editing the manuscript. MMK participated in PAH clearance methodology, measurement, and interpretation, and reviewing and editing the manuscript. DJT participated in iohexol clearance methodology, measurement, and interpretation, and reviewing and editing the manuscript. MDH participated in conceptualization and reviewing and editing the manuscript. PB contributed to methodology, conceptualization, provide resources, and participated in manuscript review and editing the manuscript. DHVR supervised and conceived the project, provided resources, and participated in methodology, project administration, and reviewing and editing the manuscript. NJN supervised and conceived the project, provided resources, and participated in methodology, project administration, conducting study visits (investigation), acquiring data, data curation, and reviewing and editing the manuscript.

## Supplementary Material

Supplemental data

ICMJE disclosure forms

Supplemental data set 1

Supplemental data set 2

Supplemental data set 3

Supplemental data set 4

Supplemental data set 5

Supplemental data set 6

Supplemental data set 7

Supporting data values

## Figures and Tables

**Table 1:**
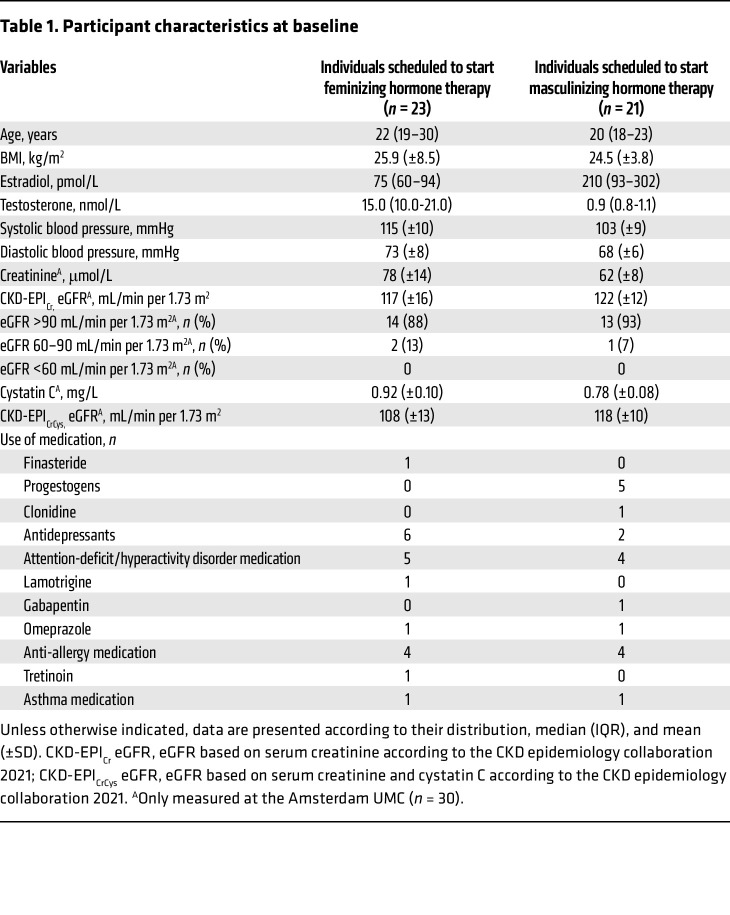
Participant characteristics at baseline

**Table 4:**
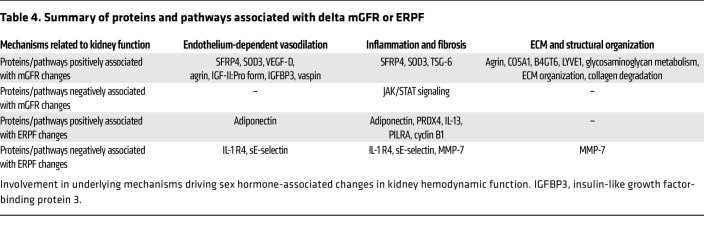
Summary of proteins and pathways associated with delta mGFR or ERPF

**Table 3:**
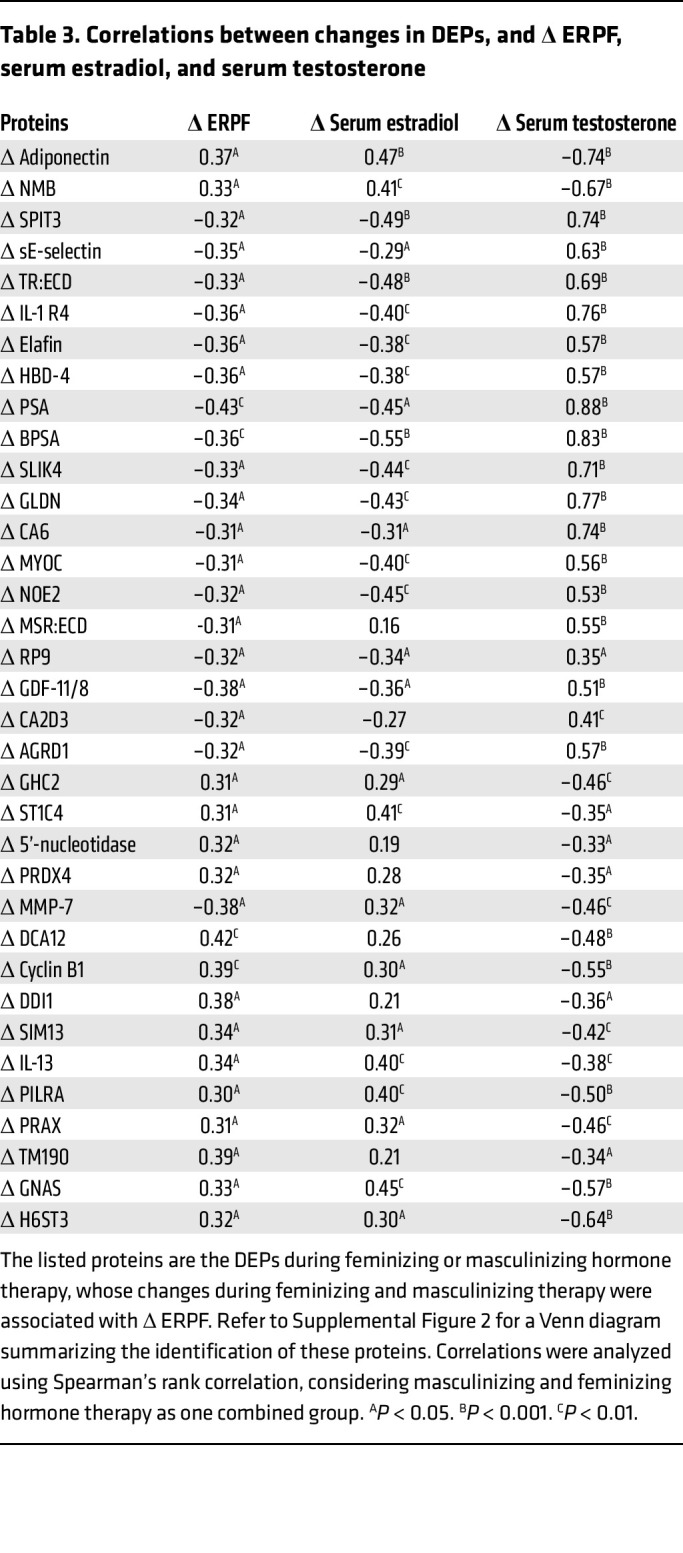
Correlations between changes in DEPs, and Δ ERPF, serum estradiol, and serum testosterone

**Table 2:**
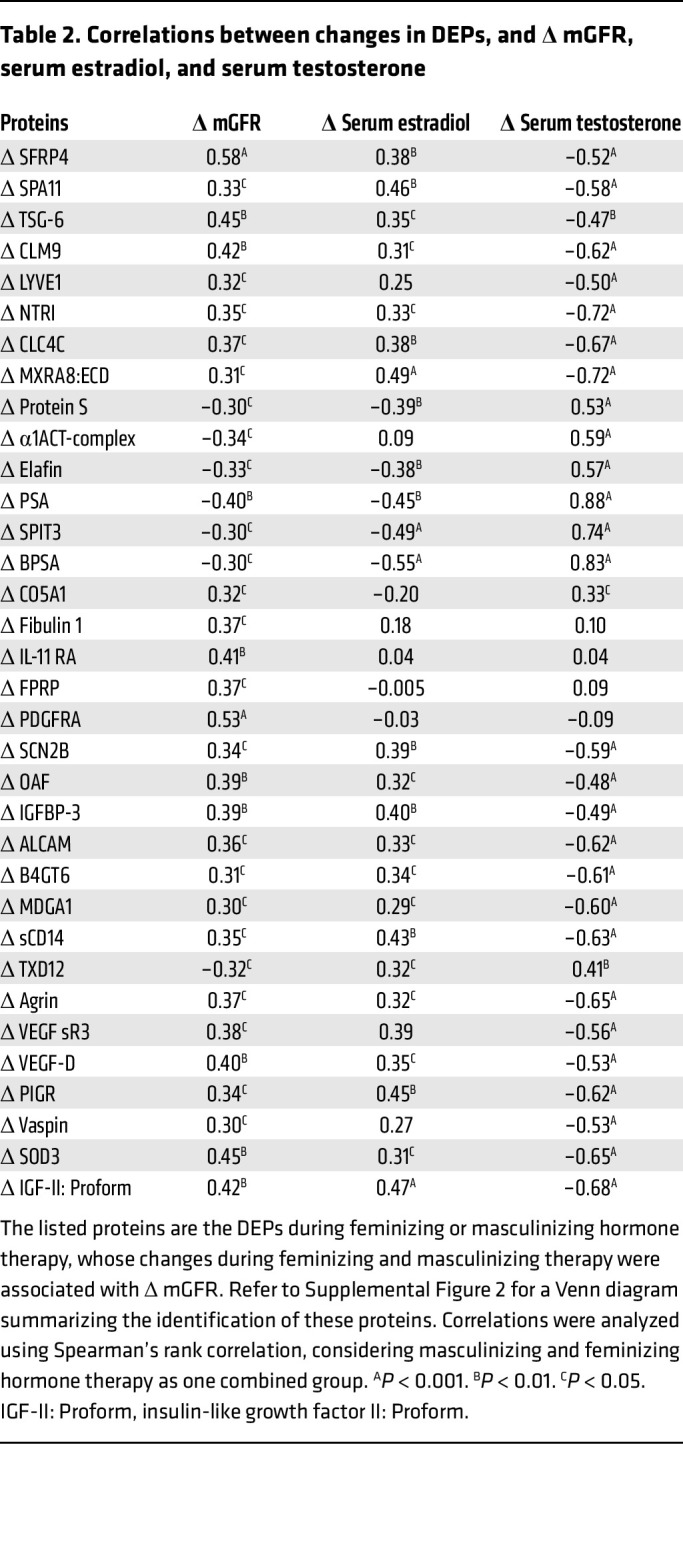
Correlations between changes in DEPs, and Δ mGFR, serum estradiol, and serum testosterone

## Version 1 04/07/2025

In-Press Preview

## Version 2 05/01/2025

Electronic publication
