# Supplementary material for: Unveiling mechanisms underlying kidney function changes during sex hormone therapy
Source: J Clin Invest. 2025 Mar 25;135(9):e190850. doi: 10.1172/JCI190850 (PMC12043095; doi:10.1172/JCI190850)
Supplement: Supplemental data [file jci-135-190850-s232.pdf]

Supplemental Appendices

Variables	Feminizing hormone therapy			Masculinizing hormone therapy		
	Baseline	*3 mo of HT*	*P-value*	*Baseline*	*3 mo of HT*	*P-value*
Estradiol, pmol/L	75 (60-94)	239 (158-301)	<0.001	210 (93-302)	158 (104-239)	0.54
Testosterone, nmol/L	15.0 (10.0-21.0)	0.6 (0.4-9.2)	<0.001	0.9 (0.8-1.1)	20.0 (9.4-29.2)	<0.001
BMI, kg/m^2	25.9 (±8.5)	26.0 (±8.3)	0.44	24.5 (±3.8)	25.0 (±4.0)	0.005
Systolic blood pressure, mmHg	114.6 (±10.1)	112.7 (±11.9)	0.25	103.3 (±8.9)	105.3 (±7.2)	0.32
Diastolic blood pressure, mmHg	72.7 (±8.2)	70.3 (±10.2)	0.089	68.0 (±5.9)	67.3	0.56
MAP, mmHg	86.7 (±8.8)	83.8 (±10.6)	0.045	79.5(±6.9)	80.2(± 6.1)	0.60

Supplemental Table 1. Sex hormone concentrations, body mass index, and blood pressure before and during three months of feminizing and masculinizing therapy. Unless otherwise indicated, data are presented according to their distribution, median (IQR), and mean (±SD). The Wilcoxon signed-rank test was used to compare serum estradiol and testosterone concentrations between baseline and three months, due to non-normal distribution of the data. For absolute changes between baseline and three-month measurements (BMI, systolic and diastolic blood pressure, and mean arterial pressure; all normally distributed), linear mixed models were used, with measurements clustered within participants and within sites. Abbreviations: HT, hormone therapy; BMI, body mass index; MAP, mean arterial pressure

**Variables**	**Feminizing hormone therapy**			**Masculinizing hormone therapy**		
	Baseline	*3 mo of HT*	*P-value*	*Baseline*	*3 mo of HT*	*P-value*
Fat-free mass, kg	55.0 (48.8-66.5)	55.6 (50.1-64.1)	0.62	43.3 (39.5-48.4)	49.2 (42.3-51.5)	<0.001
Fat mass, kg	23.8 (12.3-34.3)	24.1 (15.2-37.9)	0.44	20.3 (17.6-28.0)	17.2 (13.5-24.7)	<0.001

Supplemental Table 2. Fat-free and fat mass before and during three months of feminizing and masculinizing hormone therapy. Data are presented according to their distribution as median (IQR). To compare baseline and three months values, variables were log-transformed, and linear mixed models were applied to the log-transformed data, clustering measurements within participants and within sites. Abbreviations: HT, hormone therapy

Variables	Feminizing hormone therapy			Masculinizing hormone therapy		
	Baseline	*3 mo of HT*	*P-value*	*Baseline*	*3 mo of HT*	*P-value*
mGFR, mL/min	96.4 (89.4-111.1)	104.0 (91.7-111.5)	0.028	94.7 (84.5-99.5)	91.3 (85.9-99.4)	0.46
mGFR, mL/min per 1.73m^2	85.0 (75.2-92.4)	87.9 (77.1-96.7)	0.041	91.9 (85.3-101.9)	89.1 (83.5-95.5)	0.20
ERPF, mL/min	735 (504-845)	780 (658-896)	0.017	615 (539-646)	597 (531-669)	0.51
ERPF, mL/min per 1.73m^2	564 (476-698)	619 (561-783)	0.022	597 (522-682)	584 (554-648)	0.31
FF, %	14.8 (12.1-17.1)	12.9 (11.8-15.3)	0.21	15.5 (12.4-18.6)	16.3 (13.4-17.9)	0.89
RBF, mL/min	1301 (900-1544)	1338 (1106-1508)	0.16	1016 (849-1142)	1010 (907-1174)	0.50
RBF, mL/min per 1.73m^2	998 (815-1308)	1040 (968-1308)	0.18	976 (828-1109)	992 (896-1227)	0.75
RVR, mmHg/L/min	0.07 (0.06-0.09)	0.06 (0.05-0.08)	0.043	0.08 (0.07-0.10)	0.08 (0.07-0.09)	0.77
RVR, mmHg/L/min; corrected for BSA	0.09 (0.07-0.10)	0.08 (0.06-0.10)	0.048	0.08 (0.07-0.10)	0.08 (0.07-0.09)	0.99
P_{GLO}, mmHg	48.1 (46.8-49.9)	47.0 (45.1-48.4)	0.014	47.2 (46.0-48.3)	47.2 (46.4-48.1)	0.99
P_{GLO}, mmHg, corrected for Δ total protein			0.67			0.65
R_A, dyne × s × cm^{-5}	2309 (1920-3340)	2090 (1891-2824)	0.080	2612 (2059-2895)	2420 (1994-2861)	0.94
R_A, dyne × s × cm^{-5}, corrected for Δ total protein			0.040			0.84
R_E, dyne × s × cm^{-5}	704 (515-845)	613 (539-805)	0.73	816 (648-1028)	826 (607-930)	0.31
R_A/R_E	3.7 (3.1-4.2)	3.6 (2.4-4.3)	0.094	3.3 (2.7-4.0)	3.2 (2.9-4.1)	0.40
R_A/R_E, corrected for Δ total protein			0.050			0.36

Supplemental Table 3. mGFR and intra-kidney hemodynamic function before and during three months of feminizing and masculinizing therapy. Data are presented according to their distribution as median (IQR). To compare baseline and three months values, variables were log-transformed, and linear mixed models were applied to the log-transformed data, clustering measurements within participants and within sites. Given the significant decrease in plasma total protein concentration during feminizing hormone therapy, percentage changes in P_{GLO}, R_A, and R_A/R_E were adjusted for delta plasma total protein. Abbreviations: HT, hormone therapy; mGFR, measured glomerular filtration rate; ERPF, effective renal plasma flow; FF, filtration fraction; RBF, renal blood flow; RVR, renal vascular resistance; P_{GLO}, glomerular pressure; R_A, afferent arteriolar resistance; R_E, efferent arteriolar resistance

Variables	Feminizing hormone therapy			Masculinizing hormone therapy		
	Baseline	*3 mo of HT*	*P-value*	*Baseline*	*3 mo of HT*	*P-value*
Total protein, g/dL	7.1 (6.9-7.5)	6.8 (6.6-7.4)	0.001	7.0 (6.6-7.3)	7.0 (6.7-7.2)	0.73

Supplemental Table 4. Total protein before and during three months of feminizing and masculinizing hormone therapy. Data are presented according to their distribution as median (IQR). To compare baseline and three months values, variables were log-transformed, and linear mixed models were applied to the log-transformed data, clustering measurements within participants and within sites. Abbreviations: HT, hormone therapy

Variables	Feminizing hormone therapy (excluding spironolactone; n=18)	
	% change (95% CI)	*P-value*
mGFR, mL/min per $1.73m^2$	+3.8 (-0.2 to 7.9)	0.063
ERPF, mL/min per $1.73m^2$	+11.6 (2.4 to 21.6)	0.012
FF, %	-7.0 (-14.9 to 1.7)	0.11
RBF, mL/min per $1.73m^2$	+6.6 (-1.9 to 15.8)	0.13
RVR, mmHg/L/min; corrected for BSA	-11.0 (-19.4 to -1.6)	0.023
P_{GLO}, mmHg	-2.4 (-4.5 to -0.3)	0.023
P_{GLO}, mmHg, corrected for Δ total protein	+0.7 (-0.7 to 2.2)	0.35
R_A, dyne × s × cm^{-5},	-15.0 (-26.5 to -1.6)	0.030
R_A, dyne × s × cm^{-5}, corrected for Δ total protein	-17.3 (-31.4 to -0.3)	0.047
R_E, dyne × s × cm^{-5}	-2.8 (-11.4 to 6.7)	0.55
R_A/R_E	-12.5 (-24.0 to 0.6)	0.061
R_A/R_E, corrected for Δ total protein	-18.8 (-31.6 to -3.6)	0.017

Supplemental Table 5. Percentage change in mGFR and intra-kidney hemodynamic function before and during three months of feminizing hormone therapy after excluding participants using spironolactone (n=18). For % change, variables were log-transformed, and linear mixed models were applied to the log-transformed data, clustering measurements within participants and within sites. The resulting ratios, along with 95% confidence intervals, were back-transformed and presented as percentage changes for comparison between baseline and three-month measurements. Abbreviations: mGFR, measured glomerular filtration rate; ERPF, effective renal plasma flow; FF, filtration fraction; RBF, renal blood flow; RVR, renal vascular resistance; P_{GLO}, glomerular pressure; R_A, afferent arteriolar resistance; R_E, efferent arteriolar resistance

**Variables**	**Feminizing hormone therapy**		**Masculinizing hormone therapy**	
	% change (95% CI)		% change (95% CI)	
	Adjusted for Δ fat-free mass	Adjusted for Δ fat mass	Adjusted for Δ fat-free mass	Adjusted for Δ fat mass
mGFR, mL/min per 1.73m^2	+3.6 (0.0 to 7.4)	+3.4 (-0.1 to 7.1)	-1.4 (-7.3 to 4.9)	-2.9 (-7.6 to 2.1)
ERPF, mL/min per 1.73m^2	+10.3 (2.5 to 18.8)	+10.4 (2.4 to 18.9)	-1.1 (-9.6 to 8.3)	-3.7 (-10.6 to 3.7)

Supplemental Table 6. Percentage change in mGFR and intra-kidney hemodynamic function before and during three months of feminizing hormone therapy adjusted for delta fat-free mass and fat mass. For % change, variables were log-transformed, and linear mixed models were applied to the log-transformed data, clustering measurements within participants and within sites. The resulting ratios, along with 95% confidence intervals, were back-transformed and presented as percentage changes for comparison between baseline and three-month measurements. The model included adjustments for delta fat-free and fat mass. Abbreviations: mGFR, measured glomerular filtration rate; ERPF, effective renal plasma flow

Variables	Feminizing hormone therapy			Masculinizing hormone therapy		
	Baseline	*3 mo of HT*	*P-value*	*Baseline*	*3 mo of HT*	*P-value*
NGAL, pg/mL	2767 (1993-13405)	1932 (1513-3138)	0.009	8029 (3270-13739)	11243 (7409-24166)	0.20
NGAL, pg/mL corrected for Δ mGFR			0.024			0.13
EGF, pg/mL	1202 (826-3686)	819 (564-2487)	0.18	1250 (948-2977)	1634 (783-2852)	0.49
EGF, pg/mL corrected for Δ mGFR			0.032			0.38
UMOD, pg/mL	2503643 (1962940-4714792)	2372064 (1905937-3908150)	0.80	2732336 (1779157-4457074)	2673313 (1942371-3955739)	0.90
UMOD, pg/mL corrected for Δ mGFR			0.58			0.54
KIM-1, pg/mL	51 (33-107)	49 (25-59)	0.66	31 (19-56)	29 (18-84)	0.53
KIM-1, pg/mL corrected for Δ mGFR			0.51			0.39
MCP-1, pg/mL	18 (6-41)	11 (4-18)	0.25	6 (4-17)	8 (5-15)	0.55
MCP-1, pg/mL corrected for Δ mGFR			0.038			0.40
YKL-40, pg/mL	94 (44-192)	52 (19-148)	0.020	80 (32-214)	339 (125-582)	0.049
YKL-40, pg/mL corrected for Δ mGFR			0.005			0.050
TNFR-1, pg/mL	828 (722-955)	820 (650-900)	0.073	746 (662-826)	783 (664-990)	0.12
TNFR-1, pg/mL corrected for Δ mGFR			0.093			0.043
TNFR-2, pg/mL	4701 (3730-5957)	4675 (3814-5614)	0.54	4174 (3902-5263)	4652 (3804-5616)	0.36
TNFR-2, pg/mL corrected for Δ mGFR			0.57			0.29

Supplemental Table 7. Kidney injury biomarkers before and during three months of feminizing and masculinizing therapy. Data are presented according to their distribution as median (IQR). To compare baseline and three-month values, variables were log-transformed, and linear mixed models were applied to the log-transformed data, clustering measurements within participants and within sites. The model included adjustments for delta mGFR, and both adjusted and unadjusted results are presented. Abbreviations: HT, hormone therapy; NGAL, neutrophil gelatinase-associated lipocalin; EGF, epidermal growth factor; UMOD, uromodulin; KIM-1, kidney injury molecule-1; MCP-1, monocyte chemoattractant protein-1; YKL-40, chitinase-3-like protein 1; TNFR-1 and -2, tumor necrosis factor receptor 1 and -2.

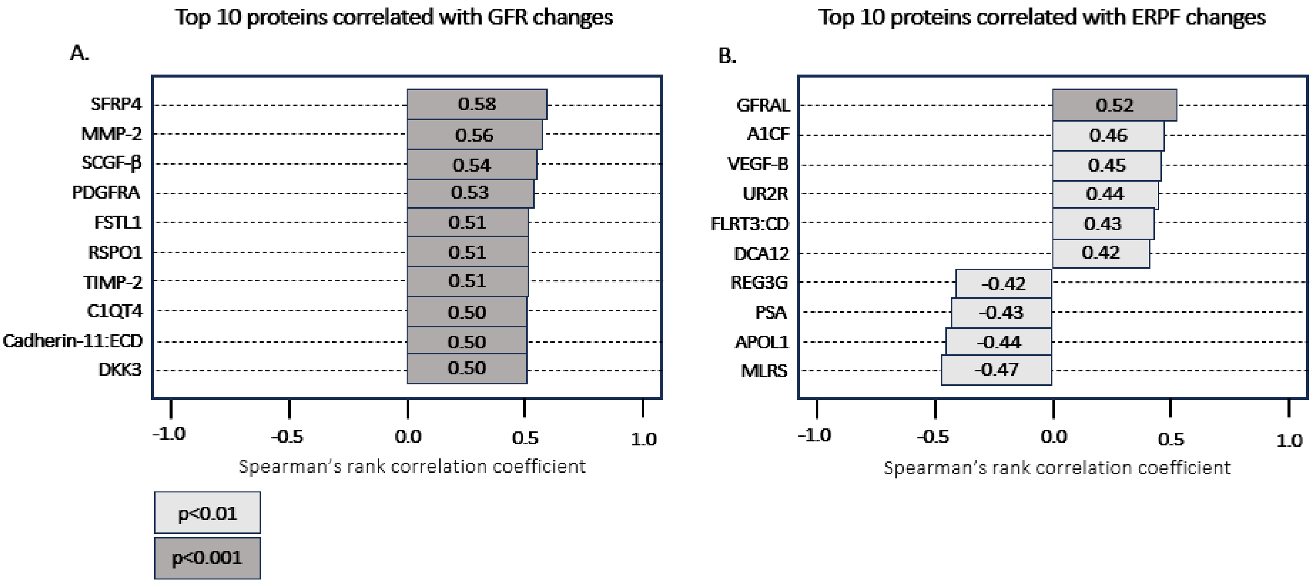

Supplemental Figure 1. Top 10 proteins that correlated with delta mGFR (A) and ERPF (B) during sex hormone therapy. Using spearman rank test, considering masculinizing and feminizing hormone therapy together as one group**.** Abbreviations: mGFR, measured glomerular filtration rate; ERPF, effective renal plasma flow; SFRP4, secreted frizzled-related protein 4; MMP-2, 72 kDa type IV collagenase; SCGF-beta, stem cell growth factor-beta; PDGFRA, platelet-derived growth factor receptor alpha; FSTL1, follistatin-related protein 1; RSPO1, R-spondin-1; TIMP-2, Metalloproteinase inhibitor 2; C1QT4, Complement C1q tumor necrosis factor-related protein 4; DKK3, Dickkopf-related protein 3; MLRS, myosin regulatory light chain 2, skeletal muscle isoform; APO L1, Apolipoprotein L1; PSA, prostate-specific antigen; REG3G, Regenerating islet-derived protein 3-gamma; GFRAL, GDNF family receptor alpha-like; A1CF, APOBEC1 complementation factor; VEGF-B, vascular endothelial growth factor B; UR2R, Urotensin-2 receptor; FLRT3:CD, Leucine-rich repeat transmembrane protein; FLRT3:Cytoplasmic domain; DCA12, DDB1- and CUL4-associated factor 12

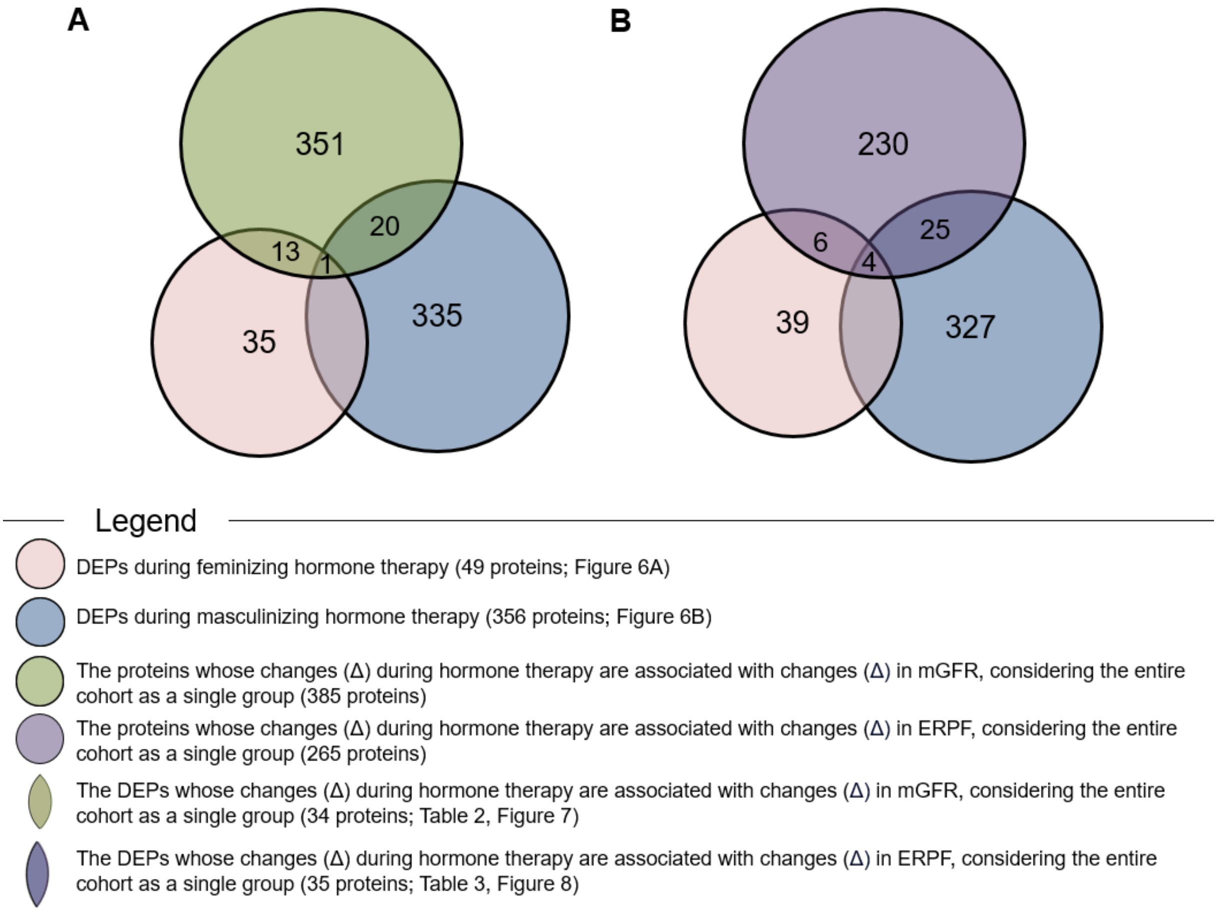

Supplemental Figure 2. Identification of DEPs whose changes during hormone therapy are associated with changes in delta mGFR or ERPF during sex hormone therapy, considering the entire cohort as a single group. A. for mGFR, B. for ERPF. Abbreviations: DEPs, differentially expressed proteins; mGFR, measured glomerular filtration rate; ERPF, effective renal plasma flow

A

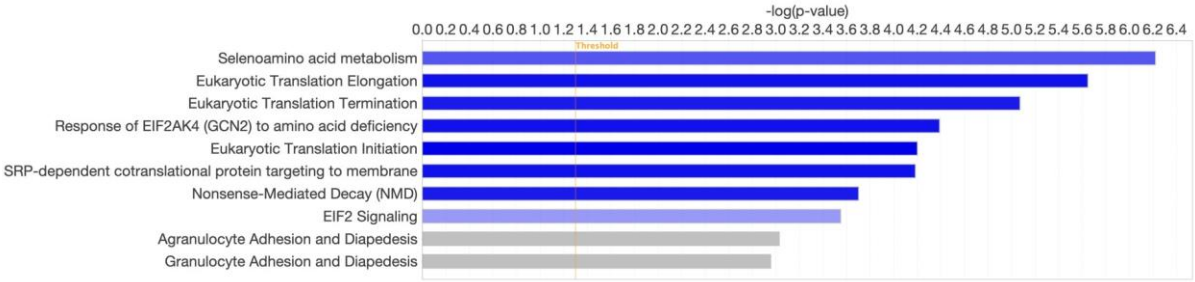

B

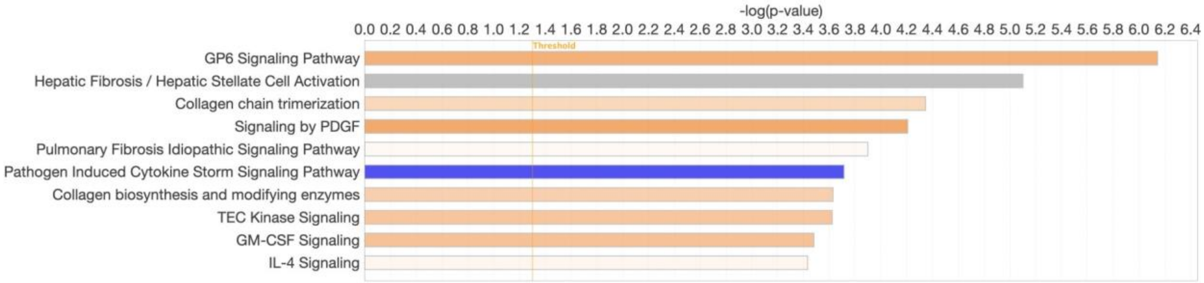

■positive z-score □z-score = 0 ■negative z-score ■no activity pattern available

Supplemental Figure 3. Z-scores of the top 10 differentially expressed pathways during feminizing (A) and masculinizing (B) hormone therapy. Abbreviations: EIF2AK4, eukaryotic translation initiation factor 2 alpha kinase 4; GCN2, general control nonderepressible 2; SRP, signal recognition particle; EIF2, eukaryotic translation initiation factor 2; GP6, glycoprotein 6; PDGF, platelet-derived growth factor; TEC, thymic epithelial cells; GM-CSF, granulocyte-macrophage colony-stimulating factor; IL-4, interleukin 4

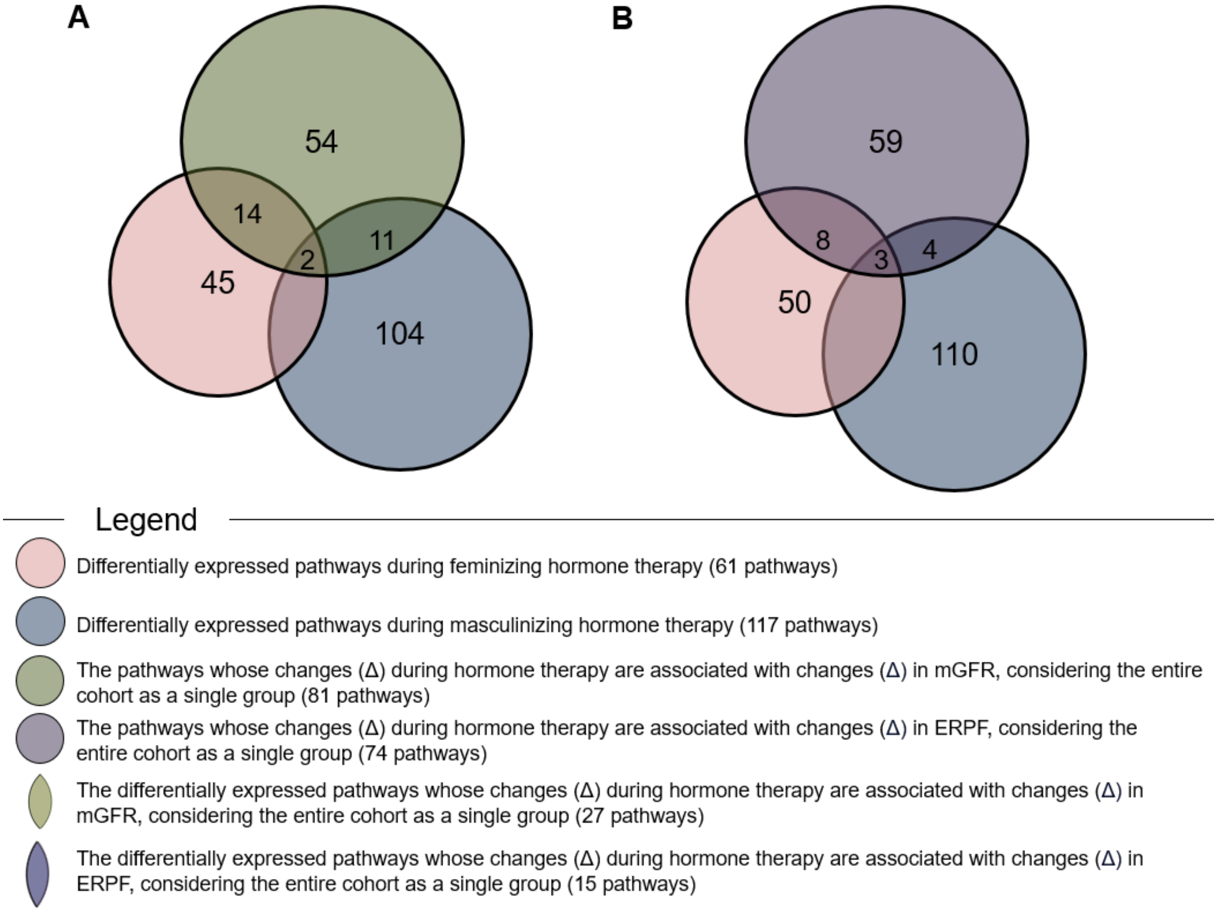

Supplemental Figure 4. Identification of differentially expressed pathways whose changes during hormone therapy are associated with delta mGFR and ERPF, considering the entire cohort as a single group. A. for mGFR, B. for ERPF. Abbreviations: mGFR, measured glomerular filtration rate; ERPF, effective renal plasma flow

Ingenuity Canonical Pathways	Z-scores (-log[p-value])	
	**During feminizing hormone therapy**	**Δ mGFR correlation**
Regulation of Insulin-like Growth Factor (IGF) transport and uptake by IGFBPs	2.0 (2.7)	3.0 (5.3)
Post-translational protein phosphorylation	1.5 (2.1)	2.7 (4.5)
Hepatic Fibrosis / Hepatic Stellate Cell Activation	# (1.7)	# (3.3)
Glycosaminoglycan metabolism	1.4 (1.7)	4.4 (2.6)
Neutrophil degranulation	0.5 (1.7)	0.2 (2.1)
LXR/RXR Activation	-1.1 (1.7)	-1.3 (1.9)
Pulmonary Healing Signaling Pathway	1.6 (1.8)	1.7 (1.7)
WNT/Ca+ pathway	0.0 (1.6)	0.8 (1.6)
Immunoregulatory interactions between a Lymphoid and a non-Lymphoid cell	1.9 (2.8)	4.2 (1.6)
Role of JAK2 in Hormone-like Cytokine Signaling	2.3 (2.2)	0.3 (1.6)
SRP-dependent cotranslational protein targeting to membrane	-3.7 (4.2)	-1.7 (1.6)
Eukaryotic Translation Initiation	-4.0 (4.2)	-3.7 (1.6)
JAK/STAT Signaling	-0.6 (1.3)	-2.5 (1.4)
EIF2 Signaling	-1.6 (3.6)	-1.9 (1.4)
Acute Phase Response Signaling	-1.7 (2.7)	-0.9 (1.3)
P2Y Purinergic Receptor Signaling Pathway	-2.7 (1.4)	-2.4 (1.3)
**Ingenuity Canonical Pathways**	**During masculinizing hormone therapy**	**Δ mGFR correlation**
Hepatic Fibrosis / Hepatic Stellate Cell Activation	# (5.1)	# (3.3)
Extracellular matrix organization	1.0 (1.4)	4.8 (3.2)
HEY1 Signaling Pathway	-0.5 (2.3)	2.0 (2.7)
Transcriptional Regulatory Network in Embryonic Stem Cells	-1.0 (1.3)	1.1 (2.2)
Th1 and Th2 Activation Pathway	# (1.3)	# (2.0)
Th1 Pathway	-2.0 (1.3)	2.1 (1.9)
Leptin Signaling in Obesity	1.7 (1.5)	0 (1.9)
Collagen degradation	1.1 (2.1)	2.3 (1.8)
Pulmonary Healing Signaling Pathway	-1.2 (1.9)	1.7 (1.7)
Pulmonary Fibrosis Idiopathic Signaling Pathway	0.2 (3.9)	2.7 (1.7)
Colorectal Cancer Metastasis Signaling	-0.1 (1.7)	-0.6 (1.6)
Role of Osteoclasts in Rheumatoid Arthritis Signaling Pathway	1.7 (2.0)	-0.4 (1.4)
FAK Signaling	-2.1 (1.8)	2.4 (1.4)

Supplemental Table 8. Z-scores of differentially expressed pathways during feminizing and masculinizing hormone therapy, and the correlation of these pathway with delta mGFR. Pathways that are listed are the ones that were significantly upregulated or downregulated during feminizing or masculinizing hormone therapy, where the changes during sex hormone therapy (considering feminizing and masculinizing therapies combined) were associated with delta mGFR; # no activity pattern available. Abbreviations: mGFR, measured glomerular filtration rate; IGFBPs, insulin-like growth factor binding proteins, LXR/RXR, liver X receptor/retinoid X receptor; SRP, signal recognition particle; EIF2, eukaryotic initiation factor 2; P2Y, purinergic receptor Y; HEY1, hairy/enhancer-of-split related with tyrosine, arginine, proline, tryptophan motif protein 1; Th1, T helper 1; Th2, T helper 2

Ingenuity Canonical Pathways	Z-scores (-log[p-value])	
	**During feminizing hormone therapy**	**Δ ERPF correlation**
Regulation of Insulin-like Growth Factor transport and uptake by IGFBPs	2.0 (2.7)	1.5 (3.2)
Hepatic Fibrosis / Hepatic Stellate Cell Activation	# (1.7)	# (2.9)
Agranulocyte Adhesion and Diapedesis	# (3.0)	# (1.9)
Activation of Matrix Metalloproteinases	-0.3 (1.5)	0.0 (1.8)
HIF1α Signaling	-0.9 (1.8)	0.6 (1.7)
Immunoregulatory interactions between a Lymphoid and a non-Lymphoid cell	1.9 (2.8)	3.1 (1.5)
Regulation of the Epithelial-Mesenchymal Transition Pathway	# (1.8)	# (1.5)
Granulocyte Adhesion and Diapedesis	# (3.0)	# (1.9)
Role of JAK2 in Hormone-like Cytokine Signaling	2.3 (2.2)	1.1 (1.4)
NAD Biosynthesis from 2-amino-3-carboxymuconate Semialdehyde	# (1.6)	# (1.3)
Post-translational protein phosphorylation	1.5 (2.1)	1.6 (1.3)
**Ingenuity Canonical Pathways**	**During masculinizing hormone therapy**	**Δ ERPF correlation**
Hepatic Fibrosis / Hepatic Stellate Cell Activation	# (5.1)	# (2.9)
HIF1α Signaling	0.7 (2.3)	0.6 (1.7)
Cardiac Hypertrophy Signaling (Enhanced)	-1.5 (1.5)	3.1 (1.7)
Regulation of the Epithelial-Mesenchymal Transition Pathway	# (1.3)	# (1.5)
Mitotic Roles of Polo-Like Kinase	-0.8 (1.4)	-1.0 (1.4)
Th1 and Th2 Activation Pathway	# (1.3)	# (1.4)
Regulation of the Epithelial Mesenchymal Transition by Growth Factors Pathway	-0.1 (2.8)	2.3 (1.3)

Supplemental Table 9. Z-scores of differentially expressed pathways during feminizing and masculinizing hormone therapy, and the correlation of these pathway with delta ERPF. Pathways that are listed are the ones that were significantly upregulated or downregulated during feminizing or masculinizing hormone therapy, where the changes during sex hormone therapy (considering feminizing and masculinizing therapies combined) were associated with delta ERPF; # no activity pattern available; Abbreviations: ERPF, effective renal plasma flow; IGFBPs, insulin-like growth factor binding protein; HIF1α, hypoxia-inducible factor 1-alpha; JAK2, janus kinase 2; NAD, nicotinamide adenine dinucleotide; Th1, T helper 1; Th2, T helper 2

Supplemental Data Files 1-7. Excel files with all proteomic data

1. Changes in proteins during feminizing hormone therapy
2. Changes in proteins during masculinizing hormone therapy
3. Changes in pathways during feminizing hormone therapy
4. Changes in pathways during masculinizing hormone therapy
5. Correlations of changes in proteins with changes in serum estradiol, changes in serum testosterone, changes in measured glomerular filtration rate, changes in effective renal plasma flow
6. Correlations of changes in pathways with changes in measured glomerular filtration rate
7. Correlations of changes in pathways with changes effective renal plasma flow

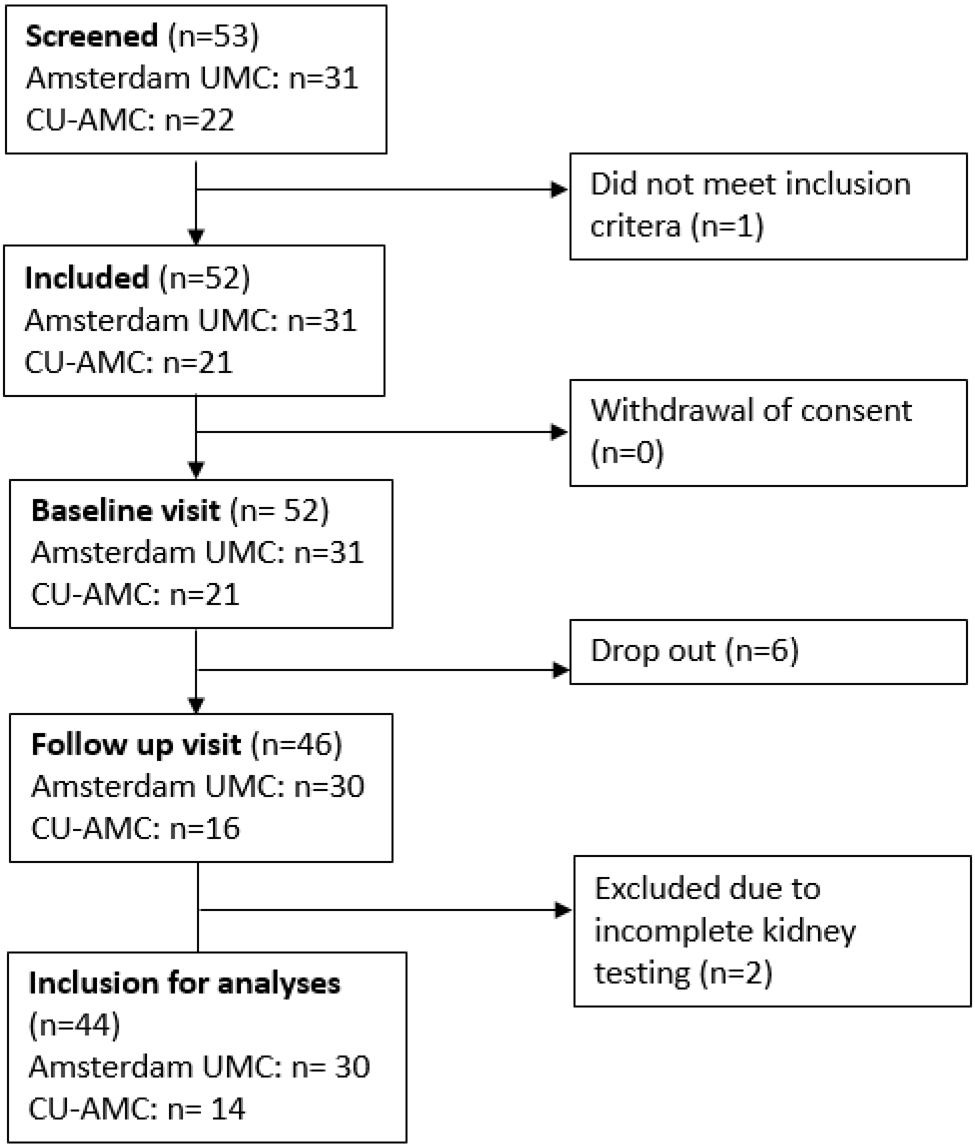

Supplemental Figure 5. Flowchart of inclusion process; Amsterdam UMC, Amsterdam university medical center; CU-AMC, University of Colorado Anschutz Medical Campus

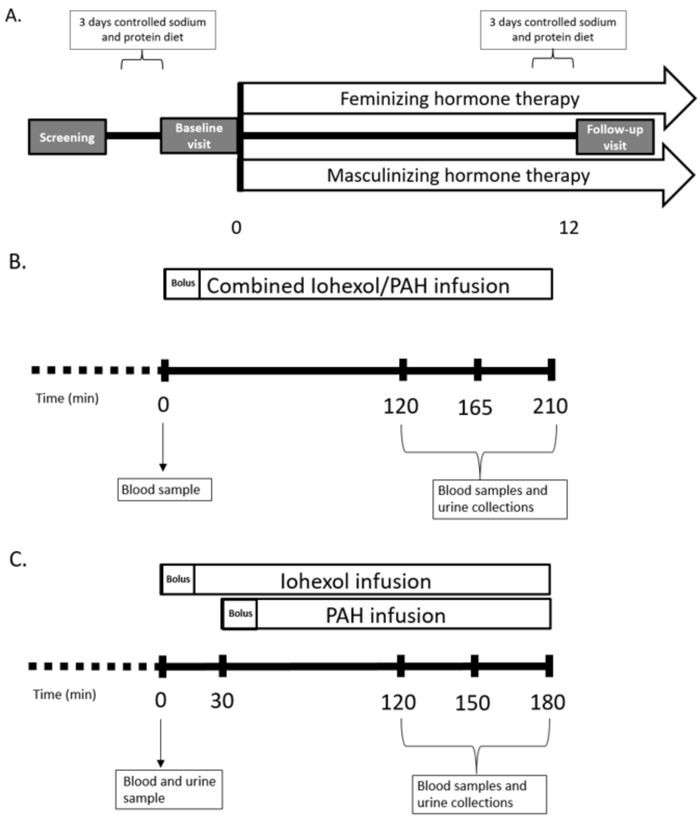

Supplemental Figure 6. Study design. (A) Study design with time in weeks. (B) Study visit design at Amsterdam UMC with time in minutes. (C) Study visit design at University of Colorado Anschutz Medical Campus with time in minutes. At least three days prior to both study visits, participants adhered to “normal” sodium (9–12 g/d) and protein (1.5–2.0 g/kg/d) diets, and were asked to refrain from vigorous physical activity and alcohol ingestion for at least 24 hours, and from consuming caffeine for at least 12 hours. Participants included in Amsterdam collected urine during a 24-hour period that ended on the night before the study visits. They were asked to fully empty their bladders, then, intravenous catheters were placed in both arms, one for infusion and one for venous blood sampling. Morning fasting blood samples were collected. Serum creatinine, estradiol, testosterone, and hematocrit were measured immediately. The remaining plasma was stored at -80°C for future analysis. After the last participant completed the study, cystatin C, total protein, TNFR-1, TNFR-2, and plasma proteomics were analyzed in batches. Subsequently, kidney function tests commenced with a weight-calculated bolus of iohexol (Omnipaque™, GE Healthcare; 36 mg/kg over 10 minutes at Amsterdam UMC and 2 minutes at CU-AMC) and PAH (4-Aminohippuric Acid Solution 20%, Basic Pharma; 3 mg/kg over 10 minutes at Amsterdam UMC and 5 minutes at CU-AMC) followed by a continuous infusion of Iohexol (15 mg/min) and PAH (12.8 mg/min for CU-AMC and 5.3 mg/min for Amsterdam UMC). In Amsterdam UMC Iohexol and PAH infusion commenced at the same time. In CU-AMC, PAH infusion commenced after 30 minutes. Blood and urine were sampled at specific time points. Systolic blood pressure, diastolic blood pressure, mean arterial pressure, and heart rate were measured during kidney testing using an automated oscillometric device (Welch Allyn Spot Vital Signs Device) positioned at the brachial artery of the nondominant arm at Amsterdam UMC and by manual cuff at CU-AMC. These measurements were taken in triplicate at intervals of 1 minute, with the final measurement used for analysis. Additionally, bioimpedance analysis (BIA; Amsterdam UMC: single-frequency bioelectrical impedance analyzer, Maltron BF-906, Maltron International, Essex, U.K.; CU-AMC: ImpediMed SFB7 single channel, tetra polar bioimpedance spectroscopy, Carlsbad, CA) was conducted to assess fat, and fat-free mass.

Supplemental Methods. Calculation of intra-kidney hemodynamics

Intra-kidney hemodynamics were estimated according to the model originally described by Gomez et al (87, 88). Filtration pressure across the glomerular capillaries (ΔPF) is calculated by the following Gomez-formula, with the gross filtration coefficient (KFG) assumed to be 0.1733 mL/sec/mmHg, P_{GLO} is 60 mmHg (given Winton's indirect estimates in the dog that glomerular pressure is roughly two-thirds of MAP), and normal glomerular oncotic pressure (πG) is 25 mmHg:

ΔPF = mGFR (mL/sec)/ KFG

πG (mmHg) is obtained from CM (plasma protein concentration within the glomerular capillaries), and calculated from TP (total protein concentration; g/dL) and FF:

CM = TP/FF * Ln (1/1 – FF)

πG = 5 * (CM – 2)

P_{GLO} was calculated by using above calculated variables and given the assumption that hydrostatic pressure in Bowman's space (PBOW) was 10 mmHg, as follows:

P_{GLO} = ΔPF + PBOW + πG

P_{GLO} = (GFR/ KFG) + 10 mmHg + [5*(TP/FF*Ln(1/ 1–FF)−2)]

Finally, in order to calculate renal vascular resistance of the afferent (R_A) and efferent (R_E) renal arteriole, we used the principles of Ohm's law, and the factor 1328 to convert to dyne•sec•cm-5:

R_A = [(MAP–PGLO/RBF]*1328

R_E = [GFR/(KFG*(RBF–GFR)]*1328

Supplemental Methods. Assays

Venous blood was drawn from the intravenous cannula using syringes, directly transferred to designated BD Vacutainer® tubes and centrifuged. Serum and plasma samples were stored at −20°C or −80°C, until batch analyses could be performed. In Amsterdam UMC, serum creatinine concentrations were measured using an enzymatic immunoassay performed on a cobas c system (Roche/Hitachi by Roche Diagnostics Mannheim, Germany). Serum cystatin C was measured using a particle-enhanced immunoturbidimetric assay on a cobas analyzer (Roche cobas 8000 module c502, Roche Diagnostics, Mannheim, Germany). EDTA- and Heparine-plasma, stored at -80ºC before assay, were used to assess Iohexol and PAH respectively. Iohexol was measured in all samples (from both sites) using liquid chromatography tandem mass spectrometry (TSQ Quantiva with UHPLC Vanquish, Thermo Fisher Scientific Waltham, MA) (89). PAH was measured in participants of Amsterdam UMC by colorimetric assay after preparation with trichloroacetic acid and indole-3-acetic acid for PAH [University Medical Center, Utrecht, The Netherlands]. In participants of CU-AMC,PAH concentrations were measured by high-performance liquid chromatography (Waters, Milford, MA).
